# Silencing PRDM14 expression by an innovative RNAi therapy inhibits stemness, tumorigenicity, and metastasis of breast cancer

**DOI:** 10.18632/oncotarget.16776

**Published:** 2017-04-01

**Authors:** Hiroaki Taniguchi, Daisuke Hoshino, Chiharu Moriya, Hitoshi Zembutsu, Nobuhiro Nishiyama, Hiroyuki Yamamoto, Kazunori Kataoka, Kohzoh Imai

**Affiliations:** ^1^ The Center for Antibody and Vaccine Therapy, Research Hospital, The Institute of Medical Science, The University of Tokyo, Tokyo 108-8639, Japan; ^2^ Cancer Biology Department, The Kanagawa Cancer Center Research Institute, Kanagawa 241-0815, Japan; ^3^ Division of Genetics, National Cancer Center Research Institute, Tokyo 104-0045, Japan; ^4^ Polymer Chemistry Division, Chemical Resources Laboratory, Tokyo Institute of Technology, Kanagawa 226-8503, Japan; ^5^ Department of Gastroenterology and Hepatology, School of Medicine, St. Marianna Medical University, Kanagawa 216-0015, Japan; ^6^ Department of Materials Engineering, Graduate School of Engineering, The University of Tokyo, Tokyo 113-8656, Japan; ^7^ The Institute of Medical Science, The University of Tokyo, Tokyo 108-8639, Japan

**Keywords:** breast cancer stem cell, cancer stemness, epigenetic alterations, nucleic acid medicine, PRDM14

## Abstract

PR domain zinc finger protein 14 (PRDM14) maintains stemness in embryonic stem cells via epigenetic mechanisms. Although PRDM14 is elevated in several cancers, it is unclear if and how PRDM14 confers stem cell-like properties and epigenetic changes to cancer cells. Here, we examined the phenotypic characteristics and epigenetic and gene expression profiles of cancer cells that differentially express PRDM14, and assessed the potential of PRDM14-targeted cancer therapy. PRDM14 expression was markedly increased in many different cancer types and correlated with poor survival of breast cancer patients. PRDM14 conferred stem cell-like phenotypes to cancer cells and regulated the expression of genes involved in cancer stemness, metastasis, and chemoresistance. PRDM14 also reduced the methylation of proto-oncogene and stemness gene promoters and PRDM14-binding regions were primarily occupied by histone H3 Lys-4 trimethylation (H3K4me3), both of which are positively correlated with gene expression. Moreover, strong PRDM14 binding sites coincided with promoters containing both H3K4me3 and H3K27me3 histone marks. Using calcium phosphate hybrid micelles as an RNAi delivery system, silencing of PRDM14 expression by chimera RNAi reduced tumor size and metastasis *in vivo* without causing adverse effects. Conditional loss of PRDM14 function also improved survival of MMTV-Wnt-1 transgenic mice, a spontaneous model of murine breast cancer. Our findings suggest that PRDM14 inhibition may be an effective and novel therapy for cancer stem cells.

## INTRODUCTION

Cancer stem cells (CSCs) comprise a small population of tumor cells that resist chemotherapeutic agents and impart oncogenic phenotypes, including metastatic capability. CSCs are therefore important therapeutic targets [1, 2]. The first CSCs to be discovered in solid tumors were tumorigenic CD44^+^/CD24^−/low^ breast cancer cells in severe combined immunodeficiency mice [3]. CSCs have subsequently been isolated from many other solid tumors [1].

PRDI-BF1 and RIZ (PR) domain zinc finger protein 14 (PRDM14) is specifically expressed in embryonic stem (ES) cells and primordial germ cells [4, 5] and, together with other factors, promotes ES cell pluripotency. PRDM14 recruits polycomb repressive complex 2 to target genes and suppresses *de novo* methyltransferases that convert the epigenome to a primed epiblast-like state [5]. PRDM14 directly binds to the proximal enhancer region of the *POU5F1* gene and upregulates OCT4 (encoded by the *POU5F1* gene) expression and colocalizes with other master regulators of pluripotency (e.g., SOX2 and NANOG) in human ES cells [6].

PRDM14 contains a PR domain homologous to the SET domain of histone lysine (Lys) methyltransferases, which regulates cell differentiation [7–9]. Epigenetic alterations such as histone modification and DNA methylation play key roles in ES cell differentiation and oncogenic pathways in cancer cells. ES cells contain many ‘poised’ bivalent chromatin domains comprising both ‘activating’ histone H3 Lys-4 trimethylation (H3K4me3) and ‘repressive’ histone H3 Lys-27 trimethylation (H3K27me3) modifications in the promoters of developmental regulatory genes [10]. When ES cells commit to a particular differentiation lineage and poised genes are activated, the ‘repressive’ H3K27me3 mark is removed and the ‘activating’ H3K4me3 mark is retained, and RNA polymerase II (Pol II) is simultaneously activated. In contrast, bivalent domains of genes associated with other lineages are silenced by retaining the H3K27me3 mark, and occurrence of H3K9me3 and DNA methylation in their promoter. In many tumors, aberrant DNA methylation is observed in the CpG island promoter around the transcription start sites (TSSs) of tumor suppressor genes, the expressions of which are silenced by DNA hypermethylation.

Previously, we showed that PRDM14 is elevated in two-thirds of breast cancers, some of which exhibit gene amplification on chromosome 8q13.3 [11]. Elevated PRDM14 expression is also associated with acute lymphatic leukemia and lung carcinoma [12, 13]. In contrast, PRDM14 is not expressed in normal differentiated tissues [11–13].

Genes that are overexpressed in cancers, such as PRDM14, may be effective targets for new therapies. Further, small interfering RNAs (siRNAs) have considerable potential as therapeutic agents for overexpressed genes. However, when administered by systemic injection, siRNAs are easily degraded by nucleases in the blood, are filtered by the kidney, accumulate poorly in target sites, and activate the innate immune system. Furthermore, siRNAs cannot readily diffuse across cell membranes and must escape from endosomes to reach their targeted mRNAs. Efforts to develop next-generation siRNA delivery strategies include modification of siRNAs and drug delivery systems (DDSs). The combination of small interfering RNA/DNA chimera (chimera RNAi) [14–16] with calcium phosphate (CaP) hybrid micelles [17] as a DDS can overcome many of the barriers encountered by standard systemic delivery systems. CaP hybrid micelles are stealth nanoparticles comprised of a CaP-nucleic acid core surrounded by a coating of polyethylene glycol (PEG)–polyanion block copolymers. The polyanion segment acts as a binding moiety with CaP nanoparticles while the PEG segment reduces non-specific interactions in the bloodstream. CaP hybrid micelles accumulate in solid tumors through enhanced permeability and retention (EPR) effects as a result of their narrow diameter distribution (30–40 nm). Further, the polyanion segment confers sensitivity to acidic pH, thereby enhancing delivery efficiency and permitting endosomal escape after endocytic internalization [17]. Therapeutic chimera RNAi can avoid off-target effects due to RISC formation of the sense strand, and has exhibited excellent stability in the bloodstream and low immunogenicity *in vivo* [14–16].

Here, we examined whether PRDM14 induces CSC-like phenotypes and influences the epigenetic state of cancer cells. Given the high PRDM14 expression in tumors and its ability to mediate pluripotency in ES cells, we hypothesized that PRDM14 contributes to CSC formation and aberrant epigenetic status in cancer. We further examined the potential of a novel breast cancer therapy that modifies *PRDM14* expression using an innovative RNAi system - chimera RNAi with CaP hybrid micelles - by systemic injection. Since PRDM14 is regulated by Wnt signaling in mouse ES cells [18,19], we validated that the therapeutic effects of silencing *PRDM14* were indeed due to PRDM14 deletion in mammary tumor virus (MMTV)-Wnt-1 mice, which ectopically express Wnt and have a high incidence of spontaneous mammary adenocarcinomas containing CSC fractions [20, 21].

## RESULTS

### PRDM14 expression in human tumors

**Figure 1:**
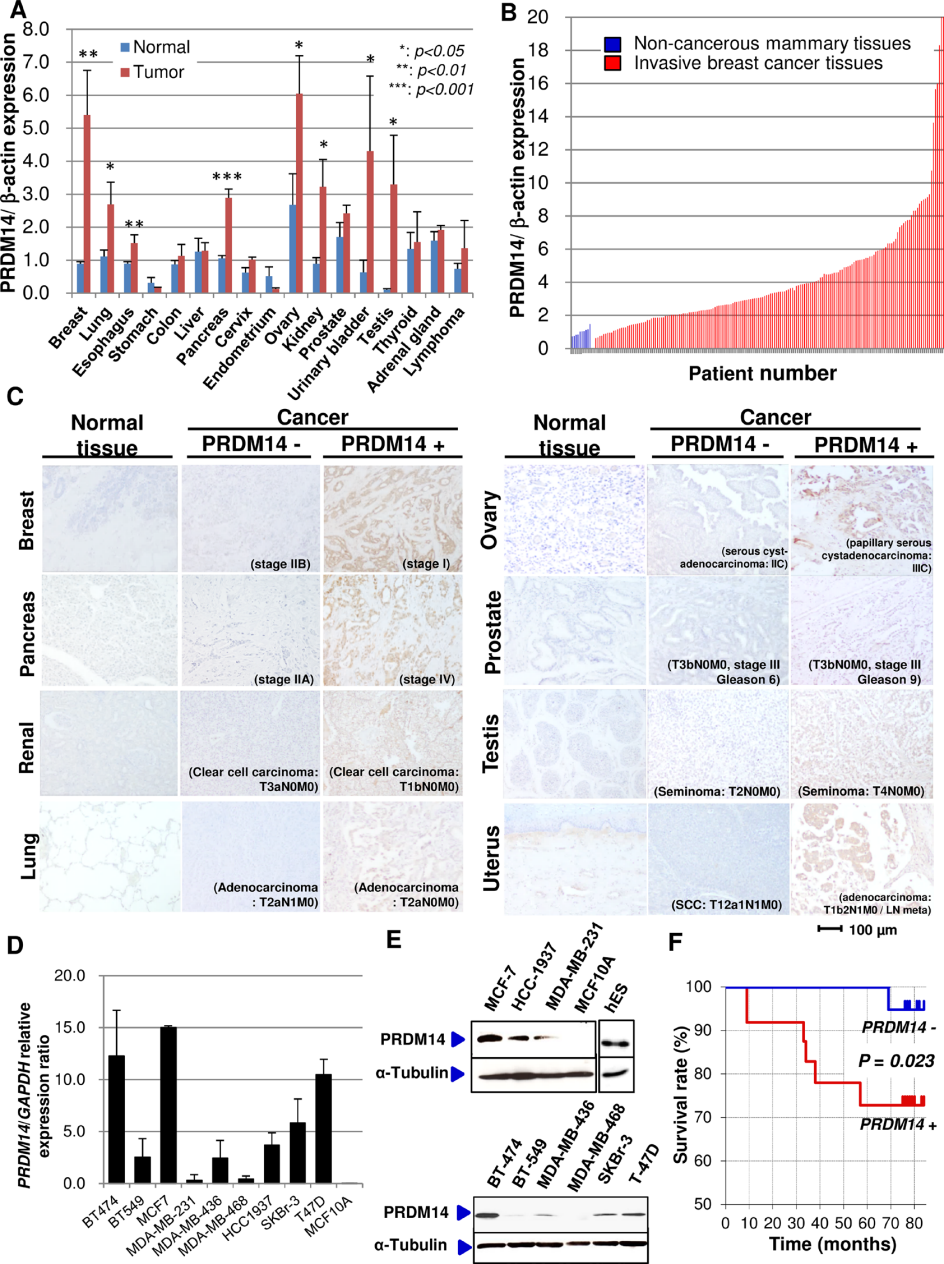
PRDM14 expression in cancer tissues (**A**) qRT-PCR analyses of *PRDM14* transcripts in different types of cancers compared to expression in respective normal tissues. Clinical samples: breast (normal/tumor [N/T], 4/24), lung (N/T, 4/19), esophageal (N/T, 3/18), stomach (N/T, 5/14), colon (N/T, 7/13), liver (N/T, 3/17), pancreatic (N/T, 5/17), cervical (N/T, 4/9), uterine (N/T, 4/17), ovarian (N/T, 3/21), renal (N/T, 5/18), prostate (N/T, 5/21), bladder (N/T, 2/22), testicular (N/T, 6/19), thyroid (N/T, 3/18), adrenal gland (N/T, 5/10), and lymphoma (N/T, 3/34). (**B**) qRT-PCR analyses of *PRDM14* transcripts in breast cancer and normal mammary tissues. Tissues from 177 invasive ductal breast carcinomas and 10 normal mammary glands. (**C**) Immunohistochemical expression of PRDM14 in different types of benign and cancerous tissues. Scale bar, 100 μm. (**D**) qRT-PCR analysis of *PRDM14* mRNA expression in breast cancer cell lines. Data are expressed as mean ± standard deviation (*n* = 3). (**E**) Immunoblot analyses of PRDM14 and α-tubulin expression in breast cancer cell lines. A lysate prepared from human embryonic stem (hES) cells served as a positive control. (**F**) Kaplan–Meier plots of the survival in breast cancer patients (*n* = 40). PRDM14–, expression scores 0–2 (*n* = 19); PRDM14+, expression scores 3–4 (*n* = 21). The *P* value from the log rank test is shown.

*PRDM14* mRNA was markedly upregulated in breast, lung, esophagus, pancreas, ovary, kidney, bladder, and testicular cancers compared to expression in the respective normal tissues (Figure 1A). *PRDM14* mRNA expression was 3-fold higher in 55.1% (97/176) of breast cancer tissues compared to normal mammary tissues obtained from 10 patients (Figure 1B).

Similarly, immunohistochemical analyses using tissue microarrays showed that PRDM14 protein was expressed in tumor tissues for breast cancer, 35.9% (n=14 out of 39 cases); lung cancer, 25.6% (10/39); pancreatic cancer, 29.3% (49/167); ovarian cancer, 37.3% (19/51); renal cancer, 38.8% (19/49); prostate cancer, 15.4% (6/39); testicular cancer, 7.0% (6/86); and cervical cancer, 18.4% (9/49). PRDM14 protein was not expressed in the corresponding normal tissues (Figure 1C).

### PRDM14 expression in breast cancer

Immunohistochemical analyses showed PRDM14 protein in 37.1% (79/213) of breast cancer patients. PRDM14 expression and patient clinicopathologic characteristics are summarized in Table 1. Tumors at stages 0, I, II, III, and IV expressed PRDM14 at frequencies of 50.0% (12/24), 28.1% (16/57), 38.9% (28/72), 40.5% (17/42), and 33.3% (6/18), respectively. There was no significant correlation between PRDM14 expression and disease stage.

**Table 1:** Correlation between human PRDM14 expression and clinicopathological characteristics of patients with breast cancer treated at the Kanagawa Cancer Research and Information Association (KCRIA)

	PRDM14- *n* (%)	PRDM14+ *n* (%)	*p* value
**Age (years)**			
**20–40**	6 (50.0%)	6 (50.0%)	*0.49* (−60 vs 61−)
**41–60**	62 (59.6%)	42 (40.4%)	
**≥ 61**	60 (61.9%)	37 (38.1%)	
**Clinical stage**			
**stage 0**	12 (50.0%)	12 (50.0%)	*0.63* (stage 0, I vs stage II~IV)
**stage I**	41 (71.9%)	16 (28.1%)	
**stage IIA**	26 (63.4%)	15 (36.6%)	
**stage IIB**	19 (61.3%)	12 (38.7%)	
**stage IIIA**	9 (56.3%)	7 (43.7%)	
**stage IIIB**	9 (64.3%)	5 (35.7%)	
**stage IIIC**	8 (66.7%)	4 (33.3%)	
**stage IV**	11 (61.1%)	7 (38.9%)	
**Estrogen receptor**			
**–**	23 (33.3%)	46 (66.7%)	*< 0.001*
**+**	97 (67.4%)	47 (32.6%)	
**Progesterone receptor**			
**–**	53 (49.5%)	54 (50.5%)	*0.95*
**+**	54 (50.9%)	52 (49.1%)	
Hercep test*			
**–**	59 (62.1%)	36 (37.9%)	*0.55* (– vs 1+~3+)
**1+**	38 (61.3%)	24 (38.7%)	
**2+**	14 (58.3%)	10 (41.7%)	
**3+**	24 (82.8%)	5 (17.2%)	
**N.D**.	2 (66.6%)	1 (33.3%)	
**CK5**			
**–**	56 (39.4%)	86 (60.6%)	*< 0.001*
**+**	1 (1.4%)	70 (98.6%)	
**CK8**			
**–**	12 (8.2%)	134 (91.8%)	*< 0.001*
**+**	45 (67.2%)	22 (32.8%)	
**Infiltrating lymphocytes**			
**–**	54 (42.2%)	74 (57.8%)	*< 0.001*
**+**	80 (94.1%)	5 (5.9%)	

*Hercep test is an FDA-approved immunohistochemical assay for the detection of HER2 protein overexpression in breast and gastric cancer.

PRDM14 expression was inversely correlated with estrogen receptor expression (*ρ =* −0.33, *P <* 0.001), a marker of breast tumor differentiation, and showed no association with progesterone receptor (*ρ =* 0.15, *P* = 0.95) or human epidermal growth factor receptor (HER)-2 expression (*ρ =* −0.09, *P* = 0.55). PRDM14^+^ breast cancer cells were positive for cytokeratin (CK)5 and negative for CK8 expression (CK5: *ρ =* 0.40, *P <* 0.001; CK8: *ρ =* −0.62, *P <* 0.001). PRDM14 expression was also significantly negatively correlated with lymphocyte infiltration in tumors (*ρ =* −0.52, *P <* 0.001): PRDM14-negative breast cancer tissues exhibited increased numbers of tumor-infiltrating cytotoxic T lymphocytes (CD3^+^CD8^+^CD16^–^). Since mammary stem cells do not express the estrogen receptor or CK8, but do express CK5/6 [22,23], our results indicate that PRDM14^+^ breast cancer cells possess features of mammary stem cell phenotypes. PRDM14 was detected at varying levels in tumorigenic breast cancer cell lines, but was not present in the non-tumorigenic MCF-10A line derived from benign proliferative breast tissue (Figure 1D, 1E).

Survival analysis on 40 tissue samples (high PRDM14 levels: n=21 samples (stage II: n=12, stage III: *n* = 9), low or undetectable levels: n=19 (stage II: *n* = 11, stage III: *n* = 8)) from breast cancer patients showed that PRDM14^+^ patients had worse prognoses than PRDM14^−^ patients (Figure 1F).

### Effect of PRDM14 on stemness phenotype

We generated three breast cancer cell lines (MCF-7, MDA-MB-231, and HCC1937) and a non-tumorigenic MCF-10A cell line overexpressing FLAG-tagged PRDM14 (Supplementary Figure 1A) to examine the functions of PRDM14 *in vitro* and *in vivo*.

**Figure 2:**
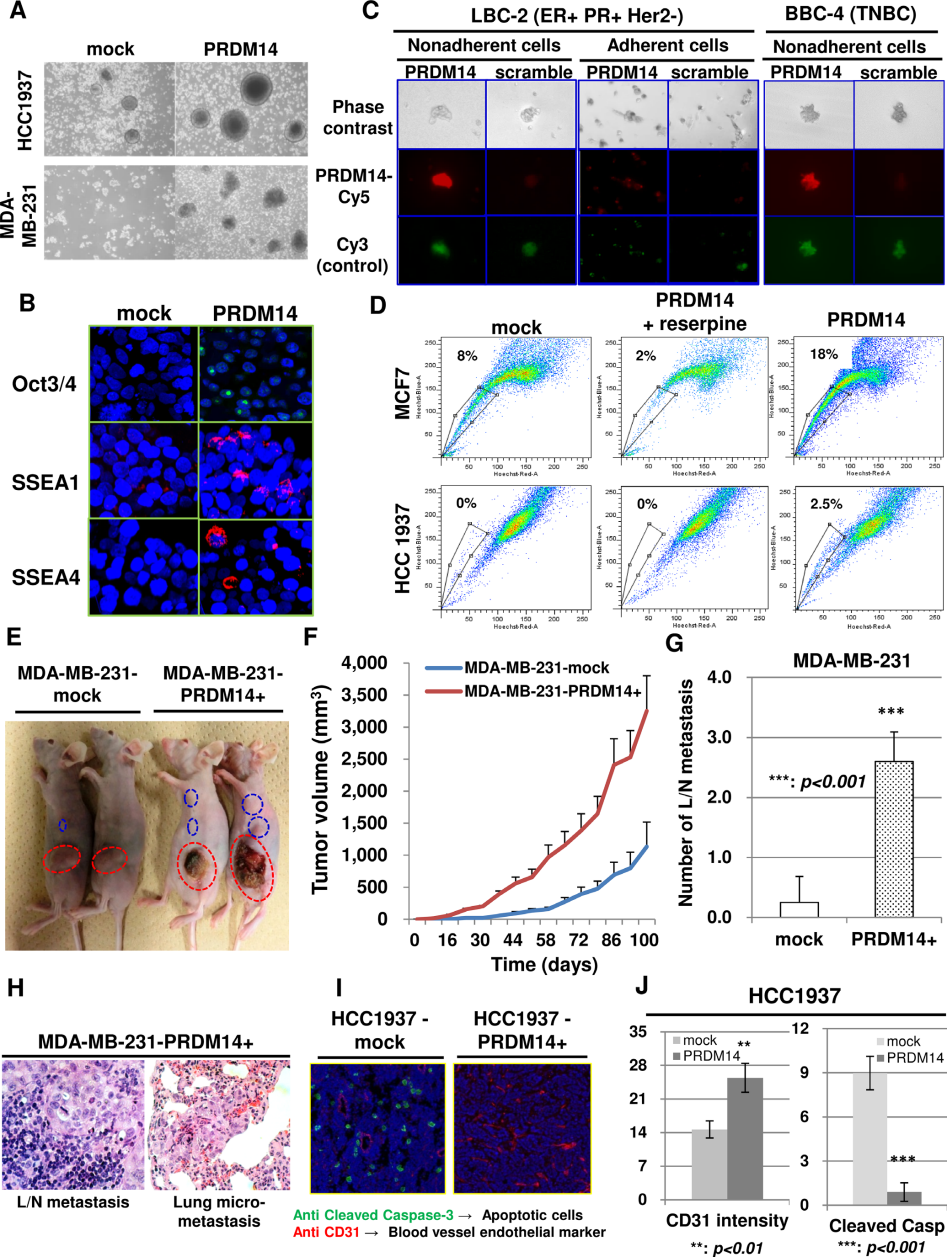
PRDM14 is required for the stemness phenotype of breast cancers (**A**) Results of a tumorsphere assay. Representative images showing increased sizes or numbers of tumorspheres after 14 days in culture. (**B**) Stemness-related markers (Oct3/4, SSEA1, and SSEA4) in tumorspheres formed by PRDM14-transfected HCC1937 cells. (**C**) *PRDM14* mRNA expression in viable cells in tumorspheres (non-adherent cells) and monolayers of primary breast cancer cells, analyzed using SmartFlare PRDM14 (Cy5) or control (Cy3) probes. (**D**) Effects of PRDM14 on the side-population fractions of PRDM14-transfected breast cancer cell lines (MCF7 and HCC1937). Reserpine, which blocks ABC transporters, was added as a negative control. (**E**) PRDM14-transfected MDA-MB-231 cells were injected into the mammary fat pad of nude mice. Images of the mice at day 100 after tumor initiation are shown. Orthografted tumors are circled with dotted red lines and lymph node metastases with dotted blue lines. (**F**) Tumor volumes determined externally using calipers of mice engrafted with PRDM14-transfected MDA-MB-231 cells (*n* = 10 for each group). (**G**) Number of lymph node metastases in mice engrafted with PRDM14-transfected MDA-MB-231 cells at day 100 after tumor initiation (*n* = 10 for each group). (**H**) Haematoxylin and eosin (H&E) staining of lungs and lymph nodes after injection of MDA-MB231-PRDM14+ cells at day 100 after tumor initiation. (**I**) Microvessel density and apoptosis was measured in frozen tumor sections by immunohistochemical analyses of CD31 and cleaved caspase-3 at day 86 after tumor initiation from mice engrafted with PRDM14-transfected HCC1937 cells in the mammary fat pad. (**J**) Microvessel density and cleaved caspase-3-positive cells in frozen tumor tissues at day 86 from mice engrafted with PRDM14-transfected HCC1937 cells in the mammary fat pad (*n* = 16). All data are expressed as mean ± SD.

PRDM14 overexpression in these cells did not increase the cell proliferation rate or colony numbers *in vitro* (Supplementary Figure 1B, 1C). We also used sphere formation assays to isolate cell subsets enriched with CSCs from solid tumors and assess the self-renewal ability of tumor cells [1]. PRDM14 overexpression promoted sphere formation and increased levels of the stem cell markers Oct3/4, SOX-2, SSEA1, and SSEA4 (Figure 2A, 2B, Supplementary Figure 1D, 1E).

We also established primary cultures of breast cancer cells derived from the initial site of invasive breast cancers from patients with luminal-type (LBC-1, LBC-2, and LBC-5) and basal-type (BBC-4) cancers. We used single-cell suspensions of each primary culture to establish secondary tumorsphere cultures and analyzed the mRNA level in viable cells (Supplementary Figure 1F). *PRDM14* expression was higher in tumorspheres than in monolayers established from the same cells (Figure 2C). Moreover, these tumorspheres were enriched in tumorigenic CD44^+^/CD24^−/low^ cells (Supplementary Figure 1G).

The side-population phenotype is characteristic of CSCs and is associated with drug resistance [24]. Side-population cells can strongly efflux Hoechst 33342 dye due to the actions of ATP-binding cassette (ABC) transporters [24, 25]. The side-population phenotype was more prominent in PRDM14-expressing cells compared to control, and was inhibited by treatment with reserpine, an ABC transporter inhibitor (Figure 2D). These data indicate that PRDM14 expression induces stemness characteristics, including tumorsphere formation, cell plasticity, and the presence of a side-population phenotype.

To assess the tumorigenic potential of cells expressing PRDM14, we orthotopically grafted nude mice with PRDM14-overexpressing MDA-MB-231 and HCC1937 cells. Mice engrafted with PRDM14 transfectants had bigger tumors compared to mock-transfected controls (Figure 2E, 2F, Supplementary Figure 1H). PRDM14-transfected cells metastasized to the lymph nodes and lungs (Figure 2E, 2G, 2H). PRDM14-expressing tumors also exhibited decreased apoptosis and higher vascular density than tumors induced by mock-transfected cells (Figure 2I, 2J).

Based on these findings, we assessed the metastatic potential of PRDM14-expressing tumor cells *in vivo* by injecting MDA-MB-231 cells through the tail vein of nude mice. Multiple large metastases were observed in the lungs of mice injected with a PRDM14 versus mock transfectant (Supplementary Figure 1I, 1J).

These results indicate that, although PRDM14 expression did not induce cell proliferation *in vitro*, PRDM14 overexpression accelerated tumor growth, metastasis, angiogenesis, and resistance to apoptosis *in vivo*.

### Effect of PRDM14 on gene expression

To determine pathway-/disease-specific genes regulated by PRDM14, we used qRT-PCR array analyses to select genes that showed > 1.5-fold differential expression in the four cell lines that overexpress PRDM14 (MDA-MB-231, MCF7, HCC1937, and MCF10A). Upregulated genes included members of the tumor necrosis factor superfamily (*TNFRSF9*, *TNFRSF11B*, and *TNFSF8*), ABC transporters (*ABCB1* and *ABCG2*), and nuclear factor (NF)-κB-related genes (*NFKB2* and *RELB*), all of which induce stemness in cancer cells [24, 26–27]. We also detected upregulation of genes involved in cancer metastasis and cancer stemness (*FOXC2*, *SNAI2*, *CXCRs*, and *IGF1*), cell stemness (*NANOG*, *POU5F1*, and *KLF4*), metalloproteinases (*MMP7*, *MMP10*, and *MMP13*), and angiogenic factors (*IL-8* and *S1PR1*) (Supplementary Table 1).

Inhibiting PRDM14 expression in MCF7 and HCC1937 cells (Supplementary Figure 2A and 2B) reduced transcription of many genes that were upregulated by PRDM14 overexpression. Downregulated genes (> 3-fold) had functions in breast cancer (*cathepsin D*, *KRT5*, and *MAPK1*), apoptosis (*TNFSF8*), drug resistance (*ABCC3*, *ABCG2*, and *EGFR*), tumor metastasis (*CD44*, *EPHB2*, *ETV4*, *IL-18*, *MMP3*, and *MMP13*), epithelial–mesenchymal transition (EMT; *Caveolin-2*, *FGFBP1*, *integrin alpha 5*, and *SERPINE1*), angiogenesis (*CTGF*, *Fibronectin 1*, *ID1*, *IL1B*, and *IL6*), and included stem cell transcription factors (*HOXA7*, *KLF4*, *PCNA*, *POU5F1*, *SOX9*, and *STAT3*) (data not shown).

PRDM14-transfected breast cancer cell lines also exhibited increased expression of oncogenic microRNAs (miRNAs) (miR-101, miR-155, miR-21, miR-221, and miR-23a) and decreased expression of tumor suppressor miRNAs (miR-128a, miR-200a/b, and miR-520f) (Supplementary Table 2).

Silencing PRDM14 reduced the expression of miRNAs upregulated in breast cancer tissues (e.g. miR-106a, miR-149, miR-18a, miR-221, miR-222, miR-224, miR-23a, miR-24, miR-27a/b, and miR-493) and increased expression of those that were downregulated (e.g. miR-15a, miR-150, miR-183, and miR-203). miR-34a, which is normally expressed at low levels by CSCs [28, 29], was elevated in PRDM14-knockdown (KD) cells (Supplementary Table 2).

qRT-PCR array analyses further detected differential expression of miRNA target genes, notably *NOTCH1*, *ABCG2*, and *ZEB1* (data available upon request).

### Epigenetic regulation of gene expression by PRDM14

DNA methylation of tumor suppressor genes is a critical step in tumor initiation and progression. Prdm14 has previously been shown to repress DNA methylation in ES cells and primordial germ cells to promote naive pluripotency and germline fate [5]. Therefore, we investigated whether PRDM14 also inhibits DNA methylation in tumorigenic and non-tumorigenic breast cells to promote cancer growth.

We profiled the methylation of 94 tumor suppressor gene promoters that exhibited aberrant DNA methylation in breast cancer tissues using EpiTect Methyl II PCR Array Human Breast Cancer. MDA-MB-231 cells stably expressing PRDM14 showed higher promoter methylation of tumor suppressor genes compared to control cells. Specifically, we detected increased methylation of the promoters of *BMP6*, *CDH1*, *EPB4IL3*, *ESR1*, *ID4*, *MUC2*, *PDLIM4*, *PER2*, *PYCARD*, *RARB*, and *TGFBI* genes. MCF-10A cells stably expressing PRDM14 exhibited higher promoter methylation of the *HS3ST3B1* gene compared to control cells. In contrast, HCC1937 and MCF7 cells stably expressing PRDM14 showed little change (<15%) in promoter methylation (Table 2).

**Table 2:** Methylation status of promoter regions of breast cancer-related genes

**Gene**	**PRDM14 transfectant 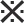 **				**% Change in DNA methylation**	
	**MDA-MB-231**		**MCF-10A**			
	**mock**	**PRDM14**	**mock**	**PRDM14**	**Hypermethylation**	
**BMP6**	29.7	70.4				90 ~ 100
**CDH1**	69.6	90.8				80 ~ 90
**EPB41L3**	73.3	95.9				70 ~ 80
**ESR1**	66.7	82.3				60 ~ 70
**GADD45A**			20.0	0.6		50 ~ 60
**HS3ST3B1**			0.1	35.7		40 ~ 50
**ID4**	20.0	89.1				30 ~ 40
**MUC2**	5.6	85.9				20 ~ 30
**PDLIM4**	29.8	67.0				10 ~ 20
**PER2**	0.8	53.3				0 ~ 10
**PROX1**	67.3	49.9				0 ~ - 10
**PYCARD**	0.0	58.9				−10 ~ −20
**RARB**	11.7	69.9				−20 ~ −40
**SYK**	93.3	62.4				−40 ~ −60
**TGFBI**	40.6	95.6				−60 ~ −80
	**PRDM14 knockdown by shRNA**					−80 ~ −90
**Gene**	**HCC1937 cell**		**MCF7 cell**			−90 ~ −100
	**compared with control (scrambled)**				**Hypomethylation**	
**CADM1**	−99.6					
**CCNA1**	−75.0					
**CTSZ**	98.9					
**CXCL12**	−24.2		−10.6			
**HS3ST3B1**	−81.6					
**KLK10**	−75.3					
**LOX**	−100.0					
**PGR**	−98.1					
**PLAGL1**	−35.0					
**PROX1**	−99.0					
**PTGS2**	−62.6					
**PYCARD**	−90.9					
**RARRES1**	99.8					
**SFRP1**			−10.9			
**SLC5A8**	−99.3					
**TGFBI**	−77.8					
**TNFRSF10D**	98.8					
**WIF1**	−20.8					
**WT1**	99.9					
**ZMYND10**	−24.5					

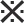
 HCC1937 cells: change in promoter methylation under 10%

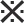
 MCF7 cells: change in promoter methylation under 10%

PRDM14-KD in HCC1937 cells with shRNA decreased promoter methylation of tumor suppressor genes (*CADM1*, *CCNA1*, *HS3ST3B1*, *CXCR12*, *KLK10*, *LOX*, *PGR*, *PLGAL1*, *PROX1*, *PTGS2*, *PYCARD*, *SLC5A8*, *TGFBI*, *WIF1*, and *ZMYND10*) but increased that of oncogenes (*CTSZ*, *RARRES1*, *TNFRSF10D*, and *WT1*). High levels of RARRES1 in clinical breast cancer samples are correlated with a more aggressive phenotype (by Ki67 positivity and overall grade) and poor patient outcome [30].

Genome-wide analysis of gene promoters with high CpG density revealed 2.7- and 5.8-fold increases in hyper- compared to hypomethylated regions in *PRDM14*-transfected MDA-MB-231 and MCF-10A cells, respectively (Supplementary Table 3). In contrast, PRDM14-KD induced similar numbers of hyper- and hypomethylated regions in MCF7 and HCC1937 cells (Supplementary Table 3). Genes with hyper- or hypomethylated regions were associated with metabolic and developmental processes and regulation of gene expression (Supplementary Figure 2C).

Taken together, these results demonstrate that PRDM14 represses promoter methylation of proto-oncogene and stemness gene promoters in PRDM14-overexpressing breast cancer cells (MCF7 and HCC1937), as it does in ES and primordial germ cells. In addition, PRDM14 enhanced DNA methylation of tumor suppressor genes in all analyzed cell lines, regardless of native PRDM14 expression levels. When the results of genome-wide methylation analyses were compared with those for downregulated genes using expression profiling data in PRDM14-expressing cells, we found that PRDM14 induced DNA methylation of the promoters of downregulated genes in PRDM14-expressing MDA-MB-231 cells.

**Figure 3:**
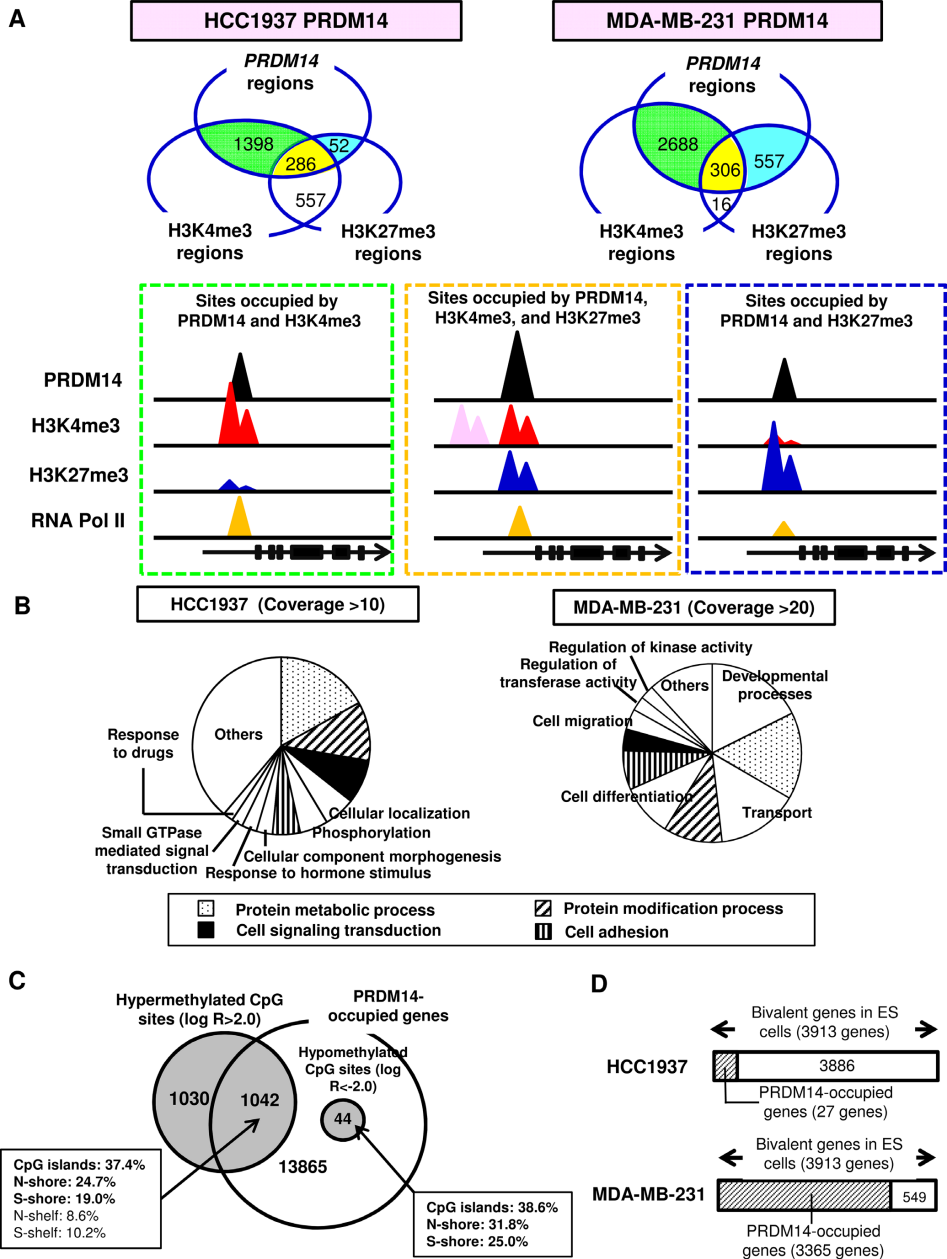
PRDM14 induces epigenetic changes (**A**) Relationships among sites occupied by PRDM14, H3K4me3, and H3K27me3 were determined using ChIP-sequencing (depth of coverage [75th percentile] > 10) in PRDM14-transfected HCC1937 and MDA-MB-231 cells. The schema of histone modification patterns of the genomes occupied by PRDM14 for those cells is shown. (**B**) Gene Ontology (GO) annotations of PRDM14-occupied genes in PRDM14-transfected HCC1937 and MDA-MB-231 cells. EASE Score, modified Fisher exact *P* value < 0.03. (**C**) Relationship between PRDM14-occupied genes and CpG sites with DNA methylation changes. Genome-wide analysis of PRDM14-transfected MDA-MB-231 cells. Percentages of promoter CpG islands and surrounding regions; i.e., shores (2-kb flanking the CpG islands) and shelves (2-kb flanking the shores). (**D**) PRDM14-occupied genes in PRDM14-transfected breast cancer cells compared with bivalent genes in hES cells.

To determine whether histone modifications required for pluripotency in ES cells are correlated with PRDM14 binding in tumor cells, we performed chromatin immunoprecipitation (ChIP)-sequencing analyses for RNA Pol II, H3K4me3, and H3K27me3 using tagged PRDM14 overexpressed in tumor cells. PRDM14-binding regions were occupied mainly by H3K4me3 and RNA Pol II (Figure 3A). Bivalent chromatin harboring both H3K4me3 and H3K27me3 were detected at distinct PRDM14-occupied loci. Other H3K4me3 peaks were detected near certain bivalent regions (Figure 3A, Supplementary Figure 2D, 2E).

Bivalent marks are common in undifferentiated cells, with one mark typically being lost during differentiation. Genes with bivalent chromatin occupied by PRDM14 were associated with cell proliferation, migration, and stemness (Supplementary Figure 2D, 2E; Supplementary Table 4). Of 286 bivalent chromatin sites occupied by PRDM14, PRDM14-KD caused 132 to switch to H3K4me3 and 8 to H3K27me3 monovalent sites in HCC1937 cells.

Using the MEME suite [31], the PRDM14-occupied consensus sequence (5′- GGTCTCTAA -3′) was identified as previously reported [5,32] (data not shown). Furthermore, PRDM14-binding peaks (false discovery rate < 0.05) were mainly distributed within 100 kb of the TSS in HCC1937 and MDA-MB-231 cells (data not shown). Gene Ontology (GO) analysis revealed significant enrichment of PRDM14-occupied genes important for development, metabolic processes, transport, protein modification, differentiation, cell signaling, and cell adhesion (Figure 3B).

In PRDM14-transfected MDA-MB-231 cells, PRDM14 co-occupied half of the hypermethylated CpG sites associated with the TSS (Figure 3C). Using the Bivalent Gene Database (BGDB) [33], we found that 86.0% and 0.7% of bivalent genes observed in human ES cells corresponded to PRDM14-occupied genes in PRDM14-overexpressing MDA-MB-231 and HCC1937 cells, respectively (Figure 3D).

### Effect of inhibiting PRDM14 expression on stem cell phenotype

**Figure 4:**
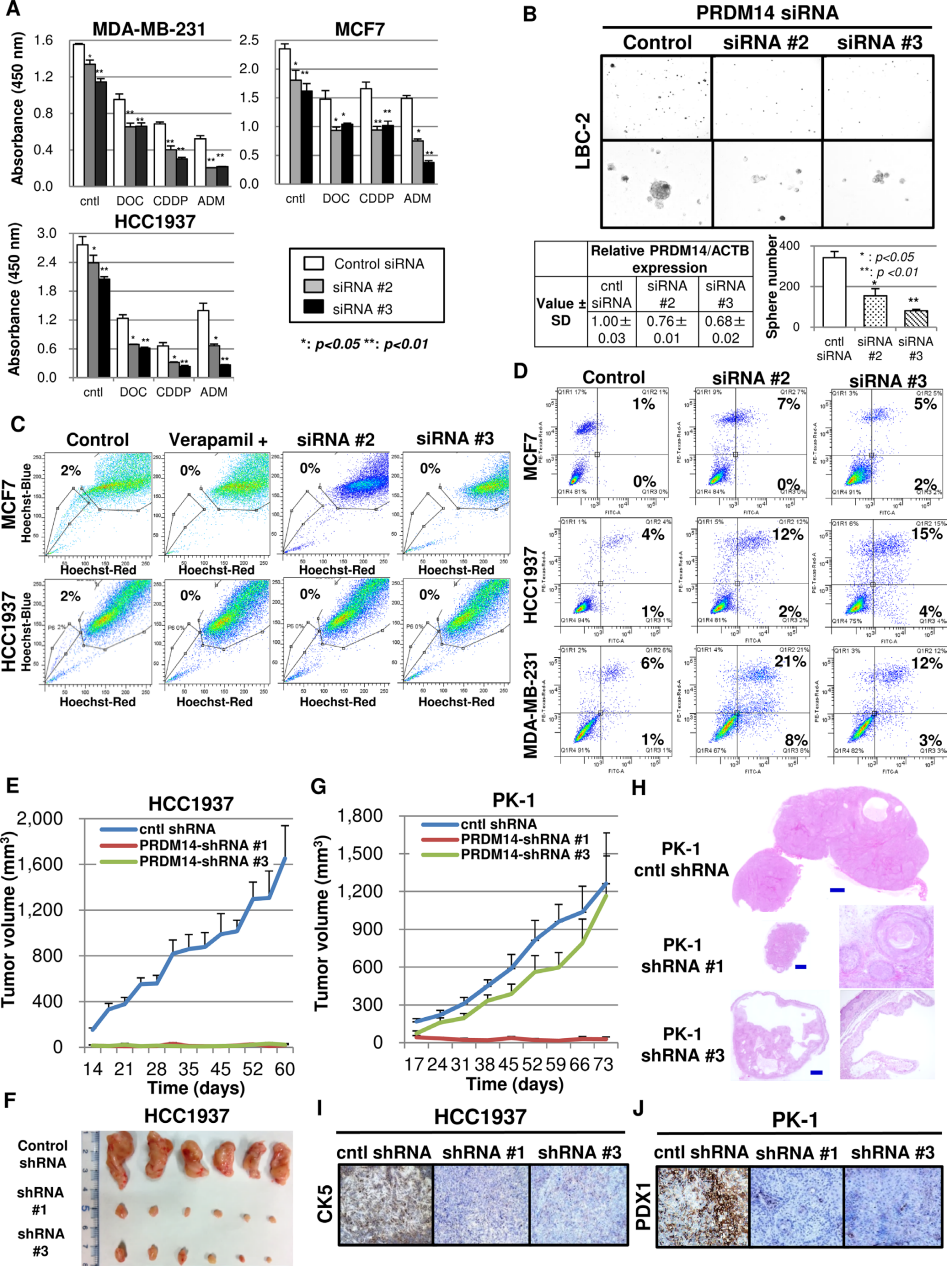
Silencing PRDM14 eliminates the stem cell phenotype and tumor growth (**A**) Proliferation of PRDM14 siRNA-transfected cancer cells cultured in the presence or absence of the indicated drugs (WST-8 assay) (*n* = 3). Control (scrambled sequence) or *PRDM14*-specific siRNA (5 nM); DOC, docetaxel (0.5 μM); CDDP, cis-diamminedichloroplatinum (25 μM); and DOX, doxorubicin (1 μM). (**B**) Tumorsphere formation by breast cancer cells in primary culture treated with control or *PRDM14*-specific siRNA (5 nM) for 72 h; 10× and 20× magnification (upper and bottom rows, respectively). (**C**) Side-population fraction of control or *PRDM14*-specific siRNA-transfected cancer cells. Verapamil, which blocks ABC transporters, was added as a negative control. (**D**) Effects of PRDM14 on apoptosis of *PRDM14*-specific siRNA-transfected breast cancer cells. Lower and upper right quadrants indicate annexin-positive early apoptotic cells and annexin/propidium iodide-positive late apoptotic cells, respectively. (**E**) Orthograft tumor growth in mice engrafted with HCC1937 cells stably transfected with control-shRNA (scrambled sequence), *PRDM14*-shRNA #1, or *PRDM14*-shRNA #3 in the mammary fat pad (*n* = 6/group). (**F**) Images of orthograft tumors from mice engrafted with HCC1937 cells stably transfected with control-shRNA (scrambled sequence), *PRDM14*-shRNA #1, or *PRDM14*-shRNA #3 in the mammary fat pad (*n* = 6/group) (day 60). (**G**) Xenograft tumor growth in nude mice engrafted with PK-1 cells (pancreatic cancer cell line) stably transfected with control-shRNA (scrambled sequence), PRDM14-shRNA #1, or PRDM14-shRNA #3 (*n* = 6/group). (**H**) H&E staining of xenograft tumors formed by PK-1 cells transfected with control shRNA, shRNA #1, or shRNA #3 at day 73 after tumor initiation; 20× (cntl shRNA and left panels for shRNA #1 and #3) and 40× magnification (right panels for shRNA #1 and #3). Scale bar, 300 μm. (**I**) CK5 expression in tumors formed by HCC1937 cells transfected with control shRNA, shRNA #1, or shRNA #3. 20× magnification. (**J**) PDX1 expression in tumors formed by PK-1 cells transfected with control shRNA, shRNA #1, or shRNA #3. 20× magnification.

Inhibiting *PRDM14* mRNA expression in MCF7 and HCC1937 cells using shRNA (Supplementary Figure 2A, 2B) did not affect cell proliferation *in vitro* (Supplementary Figure 3A). In contrast, three of seven siRNAs (#2, #3, and #5; Supplementary Table 5) inhibited PRDM14 expression in MCF7 and HCC1937 cells (Supplementary Figure 3B). Due to the 3′-UTR sequence length decrease in many siRNA-resistant cancers [34,35], we used sequences specific to the coding region of *PRDM14* (#2 and #3). These siRNAs inhibited cell proliferation of all breast cancer cell lines tested (MCF-7, MDA-MB-231, and HCC1937) *in vitro*, and suppressed cell growth in the presence of low concentrations (compared to those typically used in *in-vitro* studies) of anticancer drugs [11,36] (Figure 4A). Treating primary cultures of LBC-1, LBC-2, and LBC-5 cells with *PRDM14* siRNAs also suppressed tumorsphere formation (Figure 4B).

Downregulation of PRDM14 expression reduced side-population fractions (Figure 4C, Supplementary Figure 3C), which were increased in PRDM14-overexpressing cells. *PRDM14* siRNA-transfection into MCF7, HCC1937, and MDA-MB-231 cells induced apoptosis, which was more pronounced when the cells were exposed to anticancer drugs (Figure 4D, Supplementary Figure 3D).

### Effect of silencing PRDM14 on the growth of tumor xenografts

To determine whether the beneficial effects of PRDM14-KD observed *in vitro* could be translated *in vivo*, we examined tumor growth in nude mice engrafted with PRDM14-KD breast cancer cells. To validate this in another type of cancer with upregulation of PRDM14, we analyzed tumor growth in nude mice engrafted with PRDM14-KD pancreatic cancer (PK-1) cells (Supplementary Figure 2B). Tumors formed by KD cell lines were smaller than those induced by control cells (Figure 4E–4H). Transfecting tumors with PRDM14 abrogated the smaller tumors formed by PRDM14-KD HCC1937 cells (Supplementary Figure 3G, 3H). Furthermore, inhibiting PRDM14 expression prevented HCC1937 cells from reverting to a stem-like CD44^+^CK5^+^ phenotype (Figure 4I, Supplementary Figure 3E, 3F). Mucus production was prominent in tumors formed by shRNA#3-transfected PK-1 cells (Figure 4H). Similarly, tumors formed by PRDM14-KD PK-1 cells were differentiated and consisted of squamous or glandular epithelium with mucus production. In contrast, control PK-1 cells formed tumors with poorly differentiated PDX1-expressing cells (Figure 4H, 4J), reminiscent of the early stages of pancreatic tissue development [37].

### *In vivo* model for therapy using a PRDM14-specific siRNA

**Figure 5:**
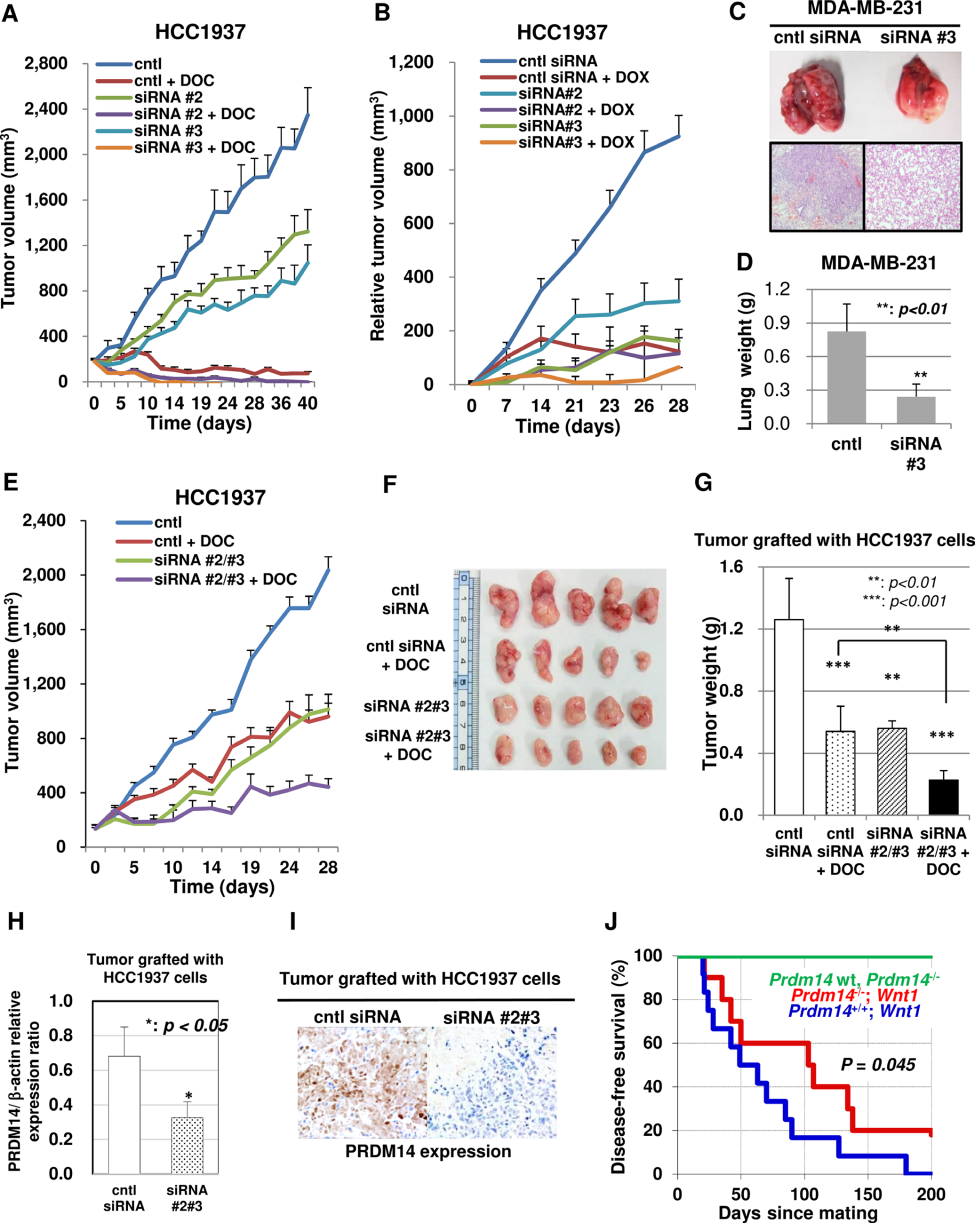
PRDM14-specific siRNA reduces the tumorigenicity of breast cancer cells *in vivo* (**A**) Intratumoral injection of the control or *PRDM14*-specific siRNA/PEI complex with or without docetaxel (DOC) reduces HCC1937 cell tumorigenicity (*n* = 6/group). (**B**) Intratumoral injection of the control or *PRDM14*-specific siRNA/PEI complex with or without doxorubicin (DOX) reduced HCC1937 cell tumorigenicity (*n* = 6/group). (**C**) Suppression of lung metastasis in mice injected intravenously with MDA-MB-231 cells treated with *PRDM14*-specific siRNA. Representative images of lungs and histological lung sections from mice treated with control (left) or PRDM14-specific siRNA (right). H&E staining; magnification, ×10. (**D**) Lung weight of mouse model for lung metastatic breast cancer (MDA-MB-231 cells) treated with *PRDM14*-specific siRNA (*n* = 8/group). (**E**) Intravenous injection (three times per week) of chimeric *PRDM14*-specific siRNA/CaP hybrid micelles with or without DOC (once per week) reduced the tumorigenicity of HCC1937 cells. Tumor volumes are shown (*n* = 8/group). (**F**) Orthografted tumor images treated with chimeric *PRDM14*-specific siRNA/CaP hybrid micelles with or without DOC at the end-point (day 28). (**G**) Orthografted tumor weights after chimeric *PRDM14*-specific siRNA/CaP hybrid micelle treatment with or without DOC (day 28) (*n* = 8/group). (**H**) *PRDM14* knockdown in orthografted tumors after chimeric *PRDM14*-specific siRNA/CaP hybrid micelle treatment was estimated using qRT-PCR (*n* = 8/group). Data are presented as mean ± SD. (**I**) Immunohistochemical expression of PRDM14 in an orthografted tumor sample after chimeric control (left) or *PRDM14*-specific siRNA/CaP hybrid micelle treatment (right). Data are presented as mean ± SD. (**J**) Kaplan-Meier curve for disease-free survival of conditional Prdm14 knock-out (cKO: Prdm14^flox/flox^; LGB-Cre) mice crossed with spontaneous model of murine breast cancer (Wnt-1 transgenic) mice. Breast tumor formation in Prdm14 cKO; Wnt-1 transgenic mice (*n* = 10; red line) compared with Prdm14^+/+^; Wnt-1 transgenic mice (*n* = 12; blue line) after parturition and lactation. Green line indicates disease-free survival of wild type mice and Prdm14 cKO mice. *p* = 0.045, Gehan-Breslow-Wilcoxon test.

We examined the effect of PRDM14-siRNA administration on tumor growth *in vivo*. We orthotopically grafted wild-type HCC1937 cells into nude mice and directly injected tumors with PRDM14-siRNA mixed with polyethyleneimine (PEI). PRDM14-specific siRNA alone decreased tumor size, an effect that was enhanced with the addition of anticancer drugs (Figure 5A, 5B). Although DOX treatment induced therapy-associated deaths in all groups, this was not observed in DOC-treated mice.

To investigate the effect of *PRDM14* siRNA on metastatic growth, MDA-MB-231 cells were injected into mice via the tail vein. After approximately 45 days, pulmonary metastases formed in the controls but not in *PRDM14* siRNA-treated mice (Figure 5C, 5D).

Chimera RNAi has many advantages over conventional siRNAs *in vivo* [14–16]. Further, CaP hybrid micelles have been used to efficiently deliver siRNA to tumors via intravenous injection [17]. Therefore, we tested this delivery method for PRDM14 chimera RNAi for cancer treatment. After the orthotopic tumor reached a predetermined size, we injected a chimera RNAi mixed with CaP hybrid micelles into the tail vein three times per week and/or a low dose of DOC intraperitoneally once each week. PRDM14-specific chimera RNAi alone decreased orthotopically grafted tumor size, which further decreased when the mice were additionally treated with DOC (Figure 5E–5G). Inhibition of PRDM14 expression in the tumor by chimera RNAi was confirmed using qRT-PCR and immunohistochemical analyses (Figure 5H, 5I). Apoptotic or necrotic areas in the tumor center were larger in the RNAi therapy group than in the control group (data not shown).

To validate the effects of PRDM14-siRNA treatment, we analyzed Prdm14 knock-out (KO) in MMTV-Wnt-1 transgenic mice, a spontaneous model of murine breast cancer. Prdm14-KO causes sterility in male and female mice due to early germ cell deficiency [4]. Therefore, we generated a conditional Prdm14 allele by introducing loxP sites flanking exon 5, which, together with exon 4, encodes the PR-domain. We crossed Prdm14^flox/+^ mice to beta-lactoglobulin promoter (LGB)-cre mice to generate a germline Prdm14 deletion only in the mammary glands. Breast tumor formation was absent in all Prdm14^flox/flox^; LGB-Cre (*n* = 22) and Prdm14^flox/flox^ (*n* = 10) mice over the course of a 200-day observation period.

To ensure that the anti-tumor effects of PRDM14-siRNA treatment were due to PRDM14 inhibition, we generated Prdm14 KO; Wnt-1 transgenic mice. Prdm14 is indirectly induced by Wnt/beta-catenin signaling [18,19]. Moreover, MMTV-Wnt-1 transgenic mice develop mammary neoplasia due to ectopic Wnt-1 production. Wnt-1 transgenic mice were used to generate Prdm14 KO; Wnt-1 mice by mating Prdm14^+/-^; LGB-cre; Wnt-1 mice with Prdm14^flox/flox^ mice. We observed breast tumor formation in both Prdm14-KO; Wnt-1 and Prdm14^flox/flox^; Wnt-1 transgenic mice after parturition and lactation (Supplementary Figure 4A). However, observational follow-up revealed that the age range of disease onset was 20–180 days (median disease-free survival time = 49 days) in Prdm14^flox/flox^; Wnt-1 transgenic mice (*n* = 12), compared to 21–138 days (median disease-free survival time = 103 days) in Prdm14-KO; Wnt-1 transgenic mice (*n* = 10). Therefore, Prdm14^flox/flox^; Wnt-1 transgenic mice have a reduced disease-free survival period compared to Prdm14-KO; Wnt-1 mice (*p* = 0.045, Gehan-Breslow-Wilcoxon test) (Figure 5J). Breast tumors formed in PRDM14-KO; Wnt-1 transgenic mice exhibited cysts with mucus production and fibrotic lesions, which were not observed in Prdm14^flox/flox^; Wnt-1 transgenic mice (Supplementary Figure 4B, 4C). Moreover, Prdm14^flox/flox^; Wnt-1 transgenic mice displayed a greater number of spontaneous lung metastases than PRDM14-KO; Wnt-1 transgenic mice (Supplementary Figure 4D–4F).

## DISCUSSION

The present study examined PRDM14 expression in cancer tissues and its effect on CSC formation by investigating whether PRDM14 induces biological properties consistent with the CSC phenotype and influences the epigenetic state of cancer cells. We showed that, in addition to the previously reported overexpression of PRDM14 in lung cancer, testicular tumors, and lymphoma, PRDM14 is also elevated in esophagus, pancreas, ovary, kidney, and bladder cancers, and is associated with poor survival of patients with breast cancer. PRDM14 conferred stem cell-like phenotypes to cancer cells and regulated the expression of genes involved in cancer stemness, metastasis, and chemoresistance. PRDM14 reduced the DNA methylation of proto-oncogene and stemness gene promoters but enhanced methylation of tumor suppressor genes in cancer cells. Administration of PRDM14 siRNA using CaP hybrid micelles reduced tumor size and metastases *in vivo*. Conditional loss of *Prdm14* function also improved survival in MMTV-Wnt-1 transgenic mice, a spontaneous model of murine breast cancer containing CSC fractions.

Although PRDM14 expression was associated with poor survival of breast cancer patients, expression was not correlated with breast cancer stage. Assuming that PRDM14 was expressed by CSCs in these tumor tissues and was involved in aberrant epigenetic changes in cancer, that PRDM14 expression was not correlated to disease stage may be explained by the heterogeneous size of the CSC population in tumors [38,39] and epigenetic changes observed in the earliest events of cancer initiation.

We showed that PRDM14 confers CSC phenotype-associated biological properties, including drug resistance, increase in the side-population fraction, rate of tumorsphere formation, and expression of stem cell markers used to identify CSCs [40]. In contrast, silencing PRDM14 decreased CSC phenotype-associated biological properties and prevented breast cancer cells from recovering stemness. These effects were not limited to breast cancer cells, as shown by our findings with PDX1, which is associated with the regulation of the earliest stages of normal pancreatic development and poor prognosis of pancreatic cancer [37]. Thus, PRDM14 is involved in maintenance of the CSC phenotype and inhibition of PRDM14 expression induces cancer cell differentiation.

PRDM14 expression did not promote cancer cell proliferation in 2D culture but enhanced tumor cell proliferation *in vivo* or in a near-*in vivo* environment, such as low attachment growth conditions. Interestingly, treatment of breast tumor spheroids, but not monolayers, with chemotherapeutic drugs induces TGF-β1 expression, mimicking tumor cell response to treatment *in vivo* [41]. Further, cancer cells in 3D culture are more similar to clinical tumor cells than cancer cells in 2D culture in terms of gene expression patterns, proliferation phenotypes, and resistance to apoptosis and chemotherapeutic drugs [41–44]. Our results indicate that PRDM14 expression is higher in tumorspheres than in monolayers of cancer cells. Tenascin C, an essential factor in the aggressiveness of pulmonary metastasis of breast cancer, is also more highly expressed in tumorspheres than in monolayer cultures of breast cancer cells [45]. Therefore, PRDM14, like tenascin C, may be required for CSC plasticity and the formation of tumorsphere initiating cells, leading to more aggressive cancer phenotypes.

Our findings also suggest that PRDM14 expression is associated with factors that inhibit immune response. We found that PRDM14 was frequently expressed by CK5^+^CK8^–^ estrogen receptor-negative breast cancer cells, and that PRDM14 prevented reversal of HCC1937 cells to a stem-like phenotype, suggesting that PRDM14 maintains breast cancer cells in an undifferentiated state. Furthermore, an inverse correlation between PRDM14 expression and lymphocyte infiltration into breast tumors indicates that PRDM14-expressing cancer cells escape immunosurveillance. This finding is supported by previous reports that breast CSCs escape T cell-mediated immunosurveillance. CD44^high^/CD24^low/neg^ breast CSCs escape NK cell-mediated surveillance [46] and express higher levels of programmed death-ligand 1 (PD-L1), which binds to the PD-1 checkpoint on immune cells [47]. Moreover, breast CSCs secrete TGF-β, which inhibits anti-tumor immune responses [48]. Therefore, we speculate that PRDM14 may prevent the detection of breast tumor cells by the immune system to worsen cancer prognosis.

To gain insight into the molecular mechanisms by which PRDM14 enhances the malignant phenotype, we investigated gene expression profiles in cells that differentially expressed PRDM14. PRDM14 regulated the expression of genes and miRNAs involved in cancer cell stemness, metastasis, and drug resistance. Moreover, silencing PRDM14 reduced the expression of CD44, which imparts cancer stemness [49], and STAT3, which is required for growth of CD44^+^CD24^–^ stem cell-like breast cancer cells [50].

PRDM14 contributes to naive pluripotency in ES cells by repressing DNA methylation [5]. We showed that PRDM14 has similar actions in breast cancer cells: PRDM14 was associated with the ‘activating’ H3K4me3 modification and reduced methylation of proto-oncogene and stemness gene promoters in breast cancer cells. Additionally, however, PRDM14 induced DNA methylation to repress several tumor suppressor genes in cancer cells, but not ES cells. In contrast, PRDM14 directly represses developmental gene expression in ES cells via the ‘repressive’ H3K27me3 mark [51].

H3K4/K27me3 modifications were found at PRDM14-occupied regions, suggesting that PRDM14 creates a bivalent histone-modification state in the promoter region of target genes. Because polycomb proteins and DNA methyltransferases are aberrantly overexpressed in cancer cells [52, 53], PRDM14 might induce DNA methylation in genome areas with bivalent chromatin to readily induce epigenetic changes. In fact, genes with promoter regions that display bivalent histone marks in ES cells are methylated in cancer cells [54]. These genes are involved in tumor progression and resistance to anticancer drugs [55]. Since PRDM14 overexpression occurs during the early stages of cancer, PRDM14 may serve as an initial trigger of epigenetic changes in tumors.

Tumor growth *in vitro* was suppressed by a *PRDM14* siRNA but was unaffected by an shRNA construct. Downregulation of Dicer or DROSHA expression is associated with an aggressive cancer phenotype [56, 57]. Downregulating Dicer inhibits the silencing effects of shRNAs but not that of siRNAs [57]. Therefore, siRNA was a better choice for *in vivo* studies for developing novel cancer therapies.

We also provided proof-of-principle analysis of an *in vivo* treatment model using CaP hybrid micelles for intravenous delivery of chimera RNAi to inhibit PRDM14 expression. This treatment caused significant tumor shrinkage and prevented metastasis without any adverse effects. Adjunct treatment with DOC further enhanced tumor shrinkage.

In our expression experiments, PRDM14 mRNA was found in normal ovarian tissue derived from non-cancerous parts of cancer tissues. PRDM14 is expressed in putative ovarian stem cells isolated from human adult ovaries [58]. Treatment with anti-PRDM14 therapy by siRNA/CaP hybrid micelles, however, should not affect the expression of PRDM14 mRNA in normal ovarian tissue since this technique specifically accumulates siRNA in targeted cancer tissues by EPR effects. Moreover, our nonclinical tests of PRDM14 siRNA with DDS in rodents and *M. fascicularis* silenced PRDM14 with no adverse effects, including on ovarian tissue, as determined by pathologic examination.

In summary, PRDM14 conferred resistance to chemotherapy and apoptosis, sphere-forming ability, enhanced vascularization, tumorigenicity and metastasis *in vivo* on breast cancer cells through a similar system to that employed in ES cells. Our results suggest that PRDM14 serves as an initial trigger for epigenetic changes and hyperdynamic plasticity in tumors via bivalent chromatin domains. Importantly, our work shows that PRDM14 may be an ideal target in many tumor types for eliminating residual cancer cells without adverse effects.

## MATERIALS AND METHODS

### Expression analyses and samples

We analyzed the RT^2^ Profiler PCR Arrays (QIAGEN, Hilden, Germany) and the Cancer Survey cDNA array or Breast Cancer cDNA array (OriGene, Rockville, MD, USA) to detect mRNA using qRT-PCR with CYBR Green and a ViiA7 System (Life Technologies, Carlsbad, CA, USA). Paraffin sections were generated from formalin-fixed tissues of 213 Japanese patients (Table 1). The Genome Ethics Committee of the University of Tokyo, and the Ethics Committee of the Kanagawa Cancer Research & Information Association (KCRIA) reviewed and approved all procedures. All samples were collected with informed consent according to the Internal Review and Ethics Boards of the Kanagawa Cancer Center (Kanagawa, Japan). PRDM14 expression and localization were determined using tissue microarrays that included tumor and normal tissues. Additional details are provided in the Supplementary Methods.

### Cell lines and cell culture

All cell lines were obtained from the American Type Culture Collection (Manassas, VA, USA) or RIKEN BRC (Tsukuba-shi, Ibaraki, Japan). The cell lines were actively passaged within 6 months of receipt. Cell lines were cultured in Dulbecco's Modified Eagle Medium (DMEM) (MDA-MB-231 and MCF7 cells) or Roswell Park Memorial Institute (RPMI) 1640 culture media (HCC1937 cells) with 10% fetal bovine serum (FBS) in 5% CO2 at 37°C.

Primary cultures from the initial site of invasive breast cancers were established from patients with luminal-type (LBC-1, LBC-2, and LBC-5) and basal-type (BBC-4) cancers. Tumor samples were washed with phosphate-buffered saline (PBS) and homogenized using Liberase TL Research Grade (Sigma-Aldrich, St. Louis, MO, USA). Cells were cultured in Ultra-Low attachment 6-well plates (Corning, NY, USA). Epidermal growth factor (EGF) and basic fibroblast growth factor (b-FGF; 20 ng/mL each) were added to 50 mL DMEM/F12 medium (Life Technologies). After the addition of 0.5 mL B27 supplement (Life Technologies), the cells were cultured at 37°C in 5% CO_2_. Further details are provided in the Supplementary Methods.

### Lentiviral transduction and siRNA transfection

Cell lines were engineered to stably express or knock down PRDM14 through lentiviral-mediated gene delivery. Lentivirus was produced by co-transfection of 293T cells with a lentiviral construct (GeneCopoeia, Rockville, MD, USA) and lentiviral packaging plasmids. Lentiviral particles were collected 48 h after transfection and filtered through a 0.45-μm filter before the addition of Polybrene (10 μg/mL). Target cells (5×10^4^ cells) were cultured with medium containing lentiviral particles for 48 hours before addition of puromycin (1 μg/mL) for selection. After selection, transfection was confirmed by mRNA and protein expression analyses.

Cancer cells were transfected with *PRDM14*-specific siRNA (5 nM) using the RNAiMAX Reagent (Life Technologies) according to the manufacturer′s protocol. The culture medium was replaced after 24 h. *In vitro* assays were performed 72 h later. For combined treatments, cis-diamminedichloroplatinum (CDDP; 25 μM), doxorubicin (DOX; 1 μM), or docetaxel (DOC; 0.5 μM) was also added to the medium 48 h later.

### Analysis of cancer cell side-population fractions

Cells were cultured at 37°C in DMEM supplemented with 5% FBS. Cells were suspended in 4 mL of 5% FBS-DMEM (1 × 10^6^ cells/mL). Reserpine (20 μM) was added to 1 mL aliquots of the cell suspensions. Cells were then incubated for 10 min at 37°C. Hoechst 33342 dye (5 μM) was added to both cell populations. The cells were incubated with shaking for 90 min at 37°C, immediately cooled on ice, centrifuged for 5 min at 300×*g*, 4°C, and resuspended in cooled 1× PBS-5% FBS solution. This procedure was repeated three times and propidium iodide was added. Analysis was performed using a FACSAria (BD Biosciences, San Jose, CA, USA). Data were analyzed using FlowLogic software (Affymetrics, Santa Clara, CA, USA).

### miRNA expression and DNA methylation profile analyses

miRNA analyses were performed using the TaqMan Array MicroRNA Card (Life Technologies) in accordance with the manufacturer's protocol. The results were analyzed using ExpressionSuite Software v1.0 (Life Technologies).

Gene methylation was evaluated by EpiTect Methyl II PCR Arrays (QIAGEN), as per the manufacturer's instructions, and Human Breast Cancer Complete Panel (QIAGEN). The Infinium Methylation 450K assay (Illumina Inc., CA, USA) was performed according to Illumina's standard protocol. Details are provided in the Supplementary Methods.

### ChIP sequencing

Breast cancer cells were transfected with a HaloTag-PRDM14 lentivirus expression vector (GeneCopoeia). PRDM14-bound genomic DNA fragments were immunoprecipitated from cell lysates with HaloLink Resin (Promega, Madison, WI, USA) and immunoprecipitated with anti-H3K4me3, anti-H3K27me3, and anti-RNAPII antibodies.

DNA fragments were processed according to the Ion PGM ChIP-sequencing protocol (Life Technologies). Ion 318 chips (Life Technologies) were used for the analysis. ChIP-DNA fragment genome mapping was performed using NextGENe software (SoftGenetics, State College, PA, USA). Details are provided in the Supplementary Methods.

### *In vivo* models

Tumor cells (1 × 10^6^ cells/mouse) were inoculated into the mammary fat pads of female nude mice (CLEA Japan, Tokyo, Japan). Tumor length and width were measured using a caliper. After tumors exceeded 100 mm^3^ in volume, siRNA and/or anticancer drug treatments were initiated. siRNA (1 mg/kg) and *in vivo*-jetPEI (Polyplus Transfection, Illkirch, France) were injected directly into the tumors three times a week. DOX (1 mg/kg) or DOC (10 mg/kg) was administered intraperitoneally once a week. Chimeric siRNA (1 mg/kg; RNAi Inc., Tokyo, Japan), a small interfering RNA/DNA chimera, was mixed with CaP hybrid micelles and injected into the tail vein three times a week. DOC (2.5 mg/kg) was administered intraperitoneally once weekly.

All animal experiments were performed with the approval of the Institutional Animal Care and Use Committee of the University of Tokyo, and adhered to the standards set for the use of mice in research. The detailed protocol is described in the Supplementary Methods.

### Statistical analysis

Mean values ± standard deviations were compared using unpaired *t*-tests. For multiple comparisons, differences were determined using one-way analysis of variance (ANOVA) followed by Tukey's post-hoc test. *P <* 0.05 was considered statistically significant.

For the Kaplan-Meier survival analyses, survival curves were compared using the logrank test for breast cancer patients or generalized Wilcoxon test for mouse cohorts.

## SUPPLEMENTARY MATERIALS FIGURES AND TABLES



## Abbreviations

3′-UTR: 3′ untranslated region
ABC: ATP-binding cassette
CaP: calcium phosphate
CDDP: cis-diamminedichloroplatinum
ChIP: chromatin immunoprecipitation
CK: cytokeratin
CSC: cancer stem cell
DMEM: Dulbecco's Modified Eagle Medium
DOC: docetaxel
DOX: doxorubicin
ES: embryonic stem
FBS: fetal bovine serum
GO: gene ontology
H3K4me3: histone H3 trimethyl Lys4
H3K27me3: histone H3 trimethyl Lys27
HER: human epidermal growth factor receptor
KD: knockdown
KO: knock-out
LGB: beta-lactoglobulin promoter
miRNA: microRNA
MMTV: mouse mammary virus tumor
PEG: polyethylene glycol
PEI: polyethyleneimine
PRDM14: PR (PRDI-BF1 and RIZ) domain zinc finger protein 14
qRT-PCR: quantitative real-time reverse transcription polymerase chain reaction
RNA Pol II: RNA polymerase II
RPMI: Roswell Park Memorial Institute
shRNA: short hairpin RNA
siRNA: small interfering RNA
TSS: transcription start site
