# Supplementary material for: Silencing PRDM14 expression by an innovative RNAi therapy inhibits stemness, tumorigenicity, and metastasis of breast cancer
Source: Oncotarget. 2017 Apr 1;8(29):46856–74. doi: 10.18632/oncotarget.16776 (PMC5564528; doi:10.18632/oncotarget.16776)
Supplement: Supplementary file 1 [file oncotarget-08-46856-s001.pdf]

Silencing PRDM14 expression by an innovative RNAi therapy inhibits stemness, tumorigenicity, and metastasis of breast cancer

Supplementary Materials

Expression analyses and samples

The qRT-PCR primer pairs (qSTAR, Origene, Rockville, MD, USA) were as follows: HP214816 (*PRDM14*), HP204660 (*ACTB*), HP205798 (*GAPDH*), HP200577 (*CD44*), and HP210404 (*CD24*). We analyzed the RT² Profiler PCR Arrays (QIAGEN, Hilden, Germany) and the Cancer Survey cDNA array or Breast Cancer cDNA array (OriGene) to detect *PRDM14* mRNA using qRT-PCR with CYBR Green and a ViiA7 System (Life Technologies, Carlsbad, CA, USA). Experiments were performed in triplicate. All PCR array data were processed using ΔΔCt-based calculations, and differentially expressed genes were identified (>1.5-fold in absolute value by duplicate analysis) by comparison with the control.

Paraffin sections were obtained from formalin-fixed tissues of 213 Japanese patients with stage 0 (24 cases), stage I (57 cases), stage II (72 cases), stage III (42 cases), and stage IV (18 cases) breast cancer (Table 1). The Genome Ethics Committee of the University of Tokyo, and the Ethics Committee of the Kanagawa Cancer Research & Information Association (KCRIA) reviewed all procedures (approval number: 24-42-0925). KCRIA samples were obtained from the Kanagawa Cancer Center Cancer Tissue Repository.

PRDM14 expression and localization were determined using tissue microarrays that included tumor and normal tissues as follows: pancreas, PA1001 and PA 1002 from US Biomax (Rockville, MD, USA) and A207V from ISU ABXIS (Seoul, Korea) (stage II, 9 cases; stage III, 147 cases; stage IV, 13 cases); breast, CBA-4; lung, CCA-4; ovary, CJ-2; kidney, CL-2; prostate, CA-4; and uterus, CZA-2 from SuperBioChips (Seoul, Korea); and testis, TE481 and TE803 from US Biomax. Samples were incubated overnight at 4°C with anti-PRDM14 primary antibody (1:100, ab192411; Abcam, Cambridge, UK) and processed as described previously [59]. Benign tissues from patients with cancer, or normal tissues, were as follows: breast (CBA-4, adjacent normal tissue, $n = 9$), lung (CCA-4, adjacent normal tissue, $n = 9$), ovary (AB-1, normal tissue, $n = 2$), kidney (CL-2, adjacent normal tissue, $n = 9$), prostate (CA-4, adjacent normal tissue, $n = 9$), and uterus (CZA-2, adjacent normal tissue, $n = 4$) from SuperBioChips; testis (TE481, normal tissue, $n = 8$ and TE803, adjacent normal tissue [$n = 10$] and normal tissue [$n = 5$]), and pancreas (PA1001, adjacent normal tissue [$n = 10$] and normal tissue [$n = 10$] and PA 1002, normal tissue [$n = 20$] from US Biomax; and A207V, adjacent normal tissue [$n = 8$] from ISU ABXIS). Immunohistochemical staining was scored by total staining intensity (high, +3; moderate, +2; weak, +1; and negative, +0) and the proportion of stained cells (≥ 50%, +2; < 50%, +1; 0% = 0). Two pathologists performed the analyses. The two scores were combined and assigned as follows: 0, negative; 1–2, weak; 3–4, positive; and 6, strongly positive.

PRDM14 protein expression was analyzed using western blotting. After sodium dodecyl sulphate-polyacrylamide gel electrophoresis (SDS-PAGE), polyvinyl difluoride (PVDF) membranes were blocked with 5% bovine serum albumin and incubated with anti-PRDM14 primary antibody (1:1000, AB4350; Millipore, Billerica, MA, USA) overnight at 4°C. PRDM14 was detected with a horseradish peroxidase (HRP)-labeled secondary antibody (GE Healthcare, Little Chalfont, UK).

Cell lines and cell culture

Cell lines (MCF7, MDA-MB-231, HCC1937, and MCF10A) were obtained from the American Type Culture Collection (Manassas, VA, USA). The PK-1 cell line was obtained from RIKEN BRC (Tsukuba-shi, Ibaraki, Japan). The cell lines were passaged actively within 6 months of receipt. Cell lines were cultured in Dulbecco's Modified Eagle Medium (DMEM) (MDA-MB-231 and MCF7 cells) or Roswell Park Memorial Institute (RPMI) 1640 (HCC1937 cells) culture media with 10% fetal bovine serum (FBS) in 5% CO_2 at 37°C. MCF-10A cells were cultured in mammary epithelial basal medium (Takara Bio, Inc., Shiga, Japan) with mammary epithelial growth medium SingleQuots (TaKaRa Bio, Inc.) and 100 ng/mL cholera toxin (Sigma-Aldrich, St. Louis, MO, USA) in 5% CO_2 at 37°C.

Primary cultures of breast cancer cells from the initial site of invasive breast cancers were established from patients with luminal-type (LBC-1, LBC-2, and LBC-5) and basal-type (BBC-4) cancers. Tumor samples were washed three times with phosphate-buffered saline (PBS) supplemented with 1× antibiotics and an antimycotic (Sigma-Aldrich) and homogenized into small fragments using Liberase TL Research Grade (Sigma-Aldrich). Cells were cultured in Ultra-Low attachment 6-well plates (Corning, NY, USA). Epidermal growth factor (EGF; 20 ng/mL) and basic fibroblast growth factor (b-FGF; 20 ng/mL) were added to 50 mL DMEM/F12 medium (Life Technologies). After the addition of 0.5 mL B27 supplement (Life Technologies), the cells were cultured at 37°C in 5% CO_2.

Lentiviral transduction and siRNA transfection

Cell lines were engineered to stably express or knockdown PRDM14 via lentiviral-mediated gene delivery. Lentivirus was produced by co-transfection of 293T cells with a lentiviral construct (GeneCopoeia, Rockville, Maryland, USA) and lentiviral packaging plasmids according to the Lenti-Pac HIV Expression Packaging kit instructions (GeneCopoeia). Lentiviral particles were collected 48 hours after transfection and filtered through a 0.45-μm filter before the addition of Polybrene (10 μg/mL). The day before transduction, target cells (5×10^4 cells) were plated in a 24-well tissue culture plate and cultured with medium containing lentiviral particles for 48 h before addition of puromycin (1 μg/mL) for selection of stably transfected cells. After selection, transfection was confirmed by mRNA and protein expression analyses.

Analyses of cell viability and apoptosis

Cell viability was evaluated using the WST-8 assay (Dojindo, Kumamoto, Japan) according to the manufacturer's protocol. Apoptosis was assessed using annexin V and propidium iodide (Abcam). After the cells were resuspended in 1× binding buffer, fluorescein isothiocyanate (FITC)-labeled annexin V reagent and propidium iodide were added. The cells were analyzed using flow cytometry. For the colony formation assay, cells were plated in triplicate on 6-well plates and maintained in media containing 10% FBS. After 14 days, the colonies were fixed with methanol, stained with 0.1% crystal violet, and the number of colonies was counted.

Tumorsphere assay

Cells were cultured in Ultra-Low attachment 6-well plates. EGF and b-FGF were added to 50 mL DMEM/F12 medium (20 ng/mL each). After the addition of 0.5 mL B27 supplement, the cells were cultured for 2 weeks and assessed for the formation of spheroids. Spheres were fixed for 20 min at room temperature in PBS containing 4% paraformaldehyde, and washed 3 times with 1% BSA in PBS for 5 min. Spheres were then permeabilized and blocked with 1% Triton X-100, 1% BSA, and 10% normal donkey serum in PBS at room temperature for 60 minutes. After blocking, spheres were incubated with fluorescent labeled antibodies (final concentration 10 μg/ml) against the stem cell markers Oct3/4, SSEA1, and SSEA4 (Human Pluripotent Stem Cell 3-Color Immunocytochemistry Kit SC021; R&D Systems, Minneapolis, MN, USA) in the dark overnight at 4°C. After washing 3 times with 1% BSA in PBS for 5 minutes, the spheres were visualized with a confocal microscope.

mRNA detection of living cells with SmartFlare probes

RNA expression in living cells was detected using a specific Smartflare Cy5-conjugated PRDM14 probe, Cy3-conjugated uptake control probe, and Cy5-conjugated scramble control probe (Merck Millipore, Massachusetts, USA). These probes are endocytosed by living cells and bind to complementary target RNA sequences, where they can be detected by fluorescence microscopy. Probes were reconstituted in 50 μl sterile nuclease free water and diluted to 1:20 in sterile PBS for use. 4 μL of diluted probes was added to 200 μL of cells in culture medium and incubated at 37°C and 5% CO_2 overnight (about 16 hours). The live cells were imaged using a fluorescence microscope (BZ-9000 BioRevo, Keyence, Tokyo, Japan).

Microarray analyses

Microarray analyses were performed with total RNA isolated from cells using a SurePrint G3 Human Gene Expression 8x60K v2 Microarray Kit (Agilent Technologies, Santa Clara, CA, USA). RNA samples had a minimum A260/A280 ratio of > 1.9 and a minimum 28S/18S ratio of > 1.6 (2100-Bioanalyzer, Agilent Technologies). Dye-swap experiments confirmed the reproducibility of the microarray data. The data from different hybridizations were scaled. A reference dataset was generated by averaging the expression of each gene across all hybridizations. Differentially expressed genes were identified using a cutoff value of $P \leq 0.01$.

miRNA expression and DNA methylation profile

miRNA analyses were performed using the TaqMan Array MicroRNA Card (Life Technologies) in accordance with the manufacturer's protocol. The results were analyzed using ExpressionSuite Software v1.0 (Life Technologies).

EpiTect Methyl II PCR Arrays (QIAGEN) were used to evaluate gene methylation as described by the manufacturer. Human Breast Cancer Complete Panel (QIAGEN) was used in this experiment. The Infinium

Methylation 450K (Illumina Inc., CA, USA) assay was performed according to the manufacturer's standard protocol. Processed methylation chips were scanned using an iScan reader (Illumina). Paired samples were processed on the same chip, and all samples were processed at the same time to avoid chip-to-chip variation. The results of genome-wide analysis of methylation sites were extracted by refinement (log ratio > 0.6, gene promoters with high CpG density).

ChIP-sequencing

Breast cancer cells were transfected with a HaloTag-PRDM14 lentivirus expression vector (GeneCopoeia, Rockville, MD, USA) and selected with puromycin. Transfection was confirmed by western blotting using the Anti-HaloTag monoclonal antibody (Promega, Madison, WI, USA). Cell lysates were used to immunoprecipitate PRDM14-bound genomic DNA fragments with HaloLink Resin (Promega). To detect histone modifications, genomic DNA fragments were immunoprecipitated with H3K4me3 and H3K27me3 antibodies (MAb-152-050 and pAb-069-050, respectively; Diagenode, Denville, NJ, USA) using the iDeal ChIP-seq Kit (Diagenode). ChIP was also performed using an anti-RNAPII antibody (AC-055-100; Diagenode).

DNA fragments were processed in accordance with the Ion PGM ChIP-sequencing protocol (Life Technologies). Three and four Ion 318 chips (Life Technologies) per sample (8 million reads per chip) were used for the analysis of transcription factors and histone modifications, respectively. ChIP-DNA fragment genome mapping was performed using NextGENe software (SoftGenetics, State College, PA, USA), and the enriched regions were detected using the sequence read concentrations.

Gene ontology

Genes that passed the filtering criteria (including the fold change cutoff) were entered into the Gene Functional Annotation Tool available at the DAVID website [60,61] using their official gene symbols. Gene ontology options GOTERM_BP_ALL were selected, and a functional annotation chart was generated. A maximum P value of 0.05 was chosen to select only significant categories.

Therapeutic model for orthotopic graft and lung metastasis

Tumor cells (1×10^6 cells/mouse) were suspended in 100 µL PBS and mixed with 100 µL Matrigel (BD Biosciences, San Jose, CA, USA) on ice. The mixture was orthotopically inoculated into the mammary fat pad of female nude mice (CLEA Japan, Tokyo, Japan) at the age of 6 weeks. After tumor formation, length and width were measured using a caliper. Tumor volume was calculated using the formula: tumor volume = length $\times$ (width)^2 $\times$ 3.14/6. After tumor tissues were formalin-fixed and embedded in paraffin, slices were stained with hematoxylin and eosin (H&E). Frozen slices were incubated overnight at 4°C with an antibody specific for cleaved caspase-3 (1:800, #9664; Cell Signaling, Danvers, MA, USA) and an anti-CD31 antibody (1:200, #55027; BD Biosciences). To create a mouse model of lung metastasis, cancer cells (1×10^6 cells/mouse) were injected into the tail vein. One month later, mice were euthanized by CO_2 inhalation and assessed for pulmonary metastases.

After tumors exceeded 100 mm^3 in volume, siRNA and/or anticancer drug treatments were initiated. siRNA (1 mg/kg) and *in vivo*-jetPEI (polyethileneimine; Polyplus Transfection, Illkirch, France) were injected directly into the tumors three times a week. siRNA (20 µg/mouse) was complexed with *in vivo*-jetPEI (2.8 µl/mouse) at an N/P ratio of 8, following the recommendations of the manufacturer (PolyPlus Transfection). Doxorubicin (DOX; 1 mg/kg) or docetaxel (DOC; 10 mg/kg) was administered intraperitoneally once a week.

To create a mouse model for treatment of pulmonary metastases, MDA-MB-231 cells (1×10^6 cells/mouse) were injected into nude mice via intravenous injection. siRNA and *in vivo*-jetPEI treatment was initiated 48h after tumor cell challenge, then administered into the orbital venous plexus under anesthesia (isoflurane) twice a week. Six weeks later, the animals were euthanized by carbon dioxide inhalation and assessed for lung weight and pulmonary metastases.

Chimera siRNA (1 mg/kg; RNAi Inc., Tokyo, Japan), a small interfering RNA/DNA chimera, was mixed with calcium phosphate (CaP) hybrid micelles (kindly provided by Dr. Kazunori Kataoka) at an N/P ratio of 5 and injected into the tail vein three times each week. DOC was administered intraperitoneally once weekly at 2.5 mg/kg.

All animal experiments were performed with the approval of the Institutional Animal Care and Use Committee of the University of Tokyo and adhere to the standards set for the use of mice in research (approval numbers: PA15-20 and PA13-02).

Conditional Prdm14 knock-out mice

All animal husbandry was carried out in accordance with protocols specified by our institutional license. We generated a conditional Prdm14 allele by introducing loxP sites flanking exon 5, which together with exon 4 encodes the PR-domain. We crossed Prdm14$^{flox/+}$ mice to beta-lactoglobulin promoter (LGB)-cre (transgenic(LGB-cre)74Acl/J) mice (Jackson Laboratory, ME, USA), generating a germline Prdm14 deletion only in mammary glands. Conditional Prdm14 knock-out (cKO: Prdm14$^{flox/flox}$; LGB-Cre) mice were crossed with Wnt-1 transgenic (B6SJL-transgenic(Wnt1)1Hev/J) mice (Jackson Laboratory) to generate Prdm14 cKO; Wnt-1 transgenic mice. Breast tumor formation was observed in both

Prdm14 cKO; Wnt-1 transgenic mice and Prdm14$^{flox/flox}$; Wnt-1 transgenic mice after parturition and lactation.

SUPPLEMENTARY REFERENCES

59. Yamamoto H, Vinitketkumnuen A, Adachi Y, Hirata T, Taniguchi H, Miyamoto N, Nosho K, Imsumran A, Fujita M, Hosokawa M, Hinoda Y, Imai K. Association of matrilysin-2 (MMP-26) expression with tumor progression and activation of MMP-9 in esophageal squamous cell carcinoma. Carcinogenesis. 2004; 25:2353–60.
60. Huang da W, Sherman BT, Lempicki RA. Bioinformatics enrichment tools: paths toward the comprehensive functional analysis of large gene lists. Nucleic Acids Res. 2009; 37:1–13.
61. Huang da W, Sherman BT, Lempicki RA. Systematic and integrative analysis of large gene lists using DAVID bioinformatics resources. Nat Protoc. 2009; 4:44–57.
62. Ajima R, Kajiya K, Inoue T, Tani M, Shiraishi-Yamaguchi Y, Maeda M, Segawa T, Furuichi T, Sutoh K, Yokota J. HOMER2 binds MYO18B and enhances its activity to suppress anchorage independent growth. Biochem Biophys Res Commun. 2007; 356:851–6.
63. Delic S, Lottmann N, Jetschke K, Reifenberger G, Riemenschneider MJ. Identification and functional validation of CDH11, PCSK6 and SH3GL3 as novel glioma invasion-associated candidate genes. Neuropathol Appl Neurobiol. 2012; 38:201–12.
64. Nguyen ST, Hasegawa S, Tsuda H, Tomioka H, Ushijima M, Noda M, Omura K, Miki Y. Identification of a predictive gene expression signature of cervical lymph node metastasis in oral squamous cell carcinoma. Cancer Sci. 2007; 98:740–6.
65. Zhou ZC, Dong QG, Fu DL, Gong YY, Ni QX. Characteristics of Notch2(+) pancreatic cancer stem-like cells and the relationship with centroacinar cells. Cell Biol Int. 2013; 37:805–11.
66. McKiernan E, McDermott EW, Evoy D, Crown J, Duffy MJ. The role of S100 genes in breast cancer progression. Tumour Biol. 2011; 32:441–50.
67. Wang L, Wang X, Liang Y, Diao X, Chen Q. S100A4 promotes invasion and angiogenesis in breast cancer MDA-MB-231 cells by upregulating matrix metalloproteinase-13. Acta Biochim Pol. 2012; 59:593–8.
68. Patarroyo M, Tryggvason K, Virtanen I. Laminin isoforms in tumor invasion, angiogenesis and metastasis. Semin Cancer Biol. 2002; 12:197–207.
69. Guo S, Bao S. srGAP2 arginine methylation regulates cell migration and cell spreading through promoting dimerization. J Biol Chem. 2010; 285:35133–41.
70. Hansel DE, Rahman A, House M, Ashfaq R, Berg K, Yeo CJ, Maitra A. Met proto-oncogene and insulin-like growth factor binding protein 3 overexpression correlates with metastatic ability in well-differentiated pancreatic endocrine neoplasms. Clin Cancer Res. 2004; 10:615258.
71. Lin L, Zhang JH, Panicker LM, Simonds WF. The parafibromin tumor suppressor protein inhibits cell proliferation by repression of the c-myc proto-oncogene. Proc Natl Acad Sci USA. 2008; 105:17420–5.
72. Sasaki T, Shiohama A, Minoshima S, Shimizu N. Identification of eight members of the Argonaute family in the human genome. Genomics. 2003; 82:323–30.
73. Wang Z, Liu N, Shi S, Liu S, Lin H. The role of PIWIL4, an Argonaute family protein, in breast cancer. J Biol Chem 2016; 291:10646-58.
74. de Bruijn DR, dos Santos NR, Kater-Baats E, Thijssen J, van den Berk L, Stap J, Balemans M, Schepens M, Merkx G, and van Kessel AG. The cancer-related protein SSX2 interacts with the human homologue of a Ras-like GTPase interactor, RAB3IP, and a novel nuclear protein, SSX2IP. Genes Chromosomes Cancer. 2002; 34:285–98.
75. Habuchi H, Tanaka M, Habuchi O, Yoshida K, Suzuki H, Ban K, Kimata K. The occurrence of three isoforms of heparan sulfate 6-O-sulfotransferase having different specificities for hexuronic acid adjacent to the targeted N-sulfoglucosamine. J Biol Chem. 2000; 275:2859–68.
76. Pellegrini L, Yu DS, Lo T, Anand S, Lee M, Blundell TL, Venkitaraman AR. Insights into DNA recombination from the structure of a RAD51-BRCA2 complex. Nature. 2002; 420:287–93.
77. Wang X, Trotman LC, Koppie T, Alimonti A, Chen Z, Gao Z, Wang J, Erdjument-Bromage H, Tempst P, Cordon-Cardo C, Pandolfi PP, Jiang X. NEDD4-1 is a proto-oncogenic ubiquitin ligase for PTEN. Cell. 2007; 128:129–39.
78. Davidson G, Wu W, Shen J, Bilic J, Fenger U, Stannek P, Glinka A, Niehrs C. Casein kinase 1 gamma couples Wnt receptor activation to cytoplasmic signal transduction. Nature. 2005; 438867–72.
79. Jauliac S, López-Rodriguez C, Shaw LM, Brown LF, Rao A, Toker A. The role of NFAT transcription factors in integrin-mediated carcinoma invasion. Nat Cell Biol. 2002; 4540–4.
80. Yu H, Lee H, Hermann A, Buettner R, Jove R. Revisiting STAT3 signalling in cancer: new and unexpected biological functions. Nat Rev Cancer. 2014; 14:736–46.
81. Samanta AK, Huang HJ, Bast RC, Jr, and Liao WS. Overexpression of MEKK3 confers resistance to apoptosis through activation of NFkappaB. J Biol Chem. 2004; 279:7576–83.
82. Abiatari I, Gillen S, DeOliveira T, Klose T, Bo K, Giese NA, Friess H, Kleeff J. The microtubule-associated protein MAPRE2 is involved in perineural invasion of pancreatic cancer cells. Int J Oncol. 2009; 35:1111–6.
83. Rodriguez-Mora OG, Lahair MM, Evans MJ, Kovacs CJ, Allison RR, Sibata CH, White KS, McCubrey JA, Franklin RA. Inhibition of the CaM-kinases augments cell death in response to oxygen radicals and oxygen radical inducing cancer therapies in MCF-7 human breast cancer cells. Cancer Biol Ther. 2006; 5:1022–30.
84. Oh JJ, Grosshans DR, Wong SG, Slamon DJ. Identification of differentially expressed genes associated with HER-2/

neu overexpression in human breast cancer cells. Nucleic Acids Res. 1999; 27:4008–17.

85. Sethi N, Yan Y, Quek D, Schupbach T, Kang Y. Rabconnectin-3 is a functional regulator of mammalian Notch signaling. J Biol Chem. 2010; 285:34757–64.

86. Tang DG, Chen YQ, Newman PJ, Shi L, Gao X, Diglio CA, Honn KV. Identification of PECAM-1 in solid tumor cells and its potential involvement in tumor cell adhesion to endothelium. J Biol Chem. 1993; 268:22883–94.

87. Nodin B, Fridberg M, Uhlén M, Jirström K. Discovery of dachshund 2 protein as a novel biomarker of poor prognosis in epithelial ovarian cancer. J Ovarian Res. 2012; 5:6.

88. Cho N, Morré DJ. Early developmental expression of a normally tumor-associated and drug-inhibited cell surface-located NADH oxidase (ENOX2) in non-cancer cells. Cancer Immunol Immunother. 2009; 58:547–52.

89. Su YC, Lin YH, Zeng ZM, Shao KN, Chueh PJ. Chemotherapeutic agents enhance cell migration and epithelial-to-mesenchymal transition through transient up-regulation of tNOX (ENOX2) protein. Biochim Biophys Acta. 2010; 1820:1744–52.

Supplementary Table 1: Expression of pathway-specific genes in breast cancer and non-tumorigenic breast cell lines transfected with the *PRDM14* vector compared with cells transfected with the empty vector

Pathway	MCF7		MDA-MB-231		HCC1937		MCF10A	
	Up	Down	Up	Down	Up	Down	Up	Down
**Apoptosis**	**CD40LG**, IL10, **TNF**, **TNFRSF11B**, **TNFRSF9**, **TNFSF8**	**CD70**, FAS	**BAG1**, **CD40LG**, **CIDEA**, FAS, **TNF**, **TNFRSF1B**, **TNFRSF11B**, **TNFRSF9**, **TNFSF8**	**BNIP3L**, **CD70**, FASLG	**BIRC3**, CD40, **CD40LG**, FASLG, **TNF**, **TNFRSF1B**, **TNFRSF11B**, **TNFRSF9,** **TNFSF8**	BCL2L10, **BNIP3L**, CIDEA	**BAG1**, BCL2L10, **BIRC3**, CD70, **CIDEA**, **TNF**, **TNFSF8**	CASP5, CD40, IL10
**Breast Cancer**	CST6, **CTSD**, **IGFBP3**, **KRT5**, **NOTCH1**, **SERPINE1**	**CCNA1**, **CDH13**, GSTP1, MUC1, TFF3	**CTSD**, GSTP1, **IL6**, **KRT5**, MUC1, **NOTCH1**, **SERPINE1**	**CCNA1**, CDH1, **CDH13**, CST6, **FOXA1**, **SFRP1**, SLIT2, TFF3	CDH1, **IGFBP3**, **IL6**, **KRT5**, **NOTCH1**, **SERPIN1**, SLIT2, TFF3	**SFRP1**	MUC1, TFF3	**FOXA1**
**Cancer Drug Resistance**	**ABCB1**, **ABCG2**, CYP1A2, CYP2B6	CYP2C8, CYP3A4	CYP2C8, CYP3A5, **NFKB2**, **RELB**	—	**ABCB1**, **ABCG2**, CYP1A2, CYP2B6, **NFKB2**, **RELB**	CYP3A4, SULT1E1	CYP3A4, CYP3A5, SULT1E1	ABCB1, ABCG2, CYP2C8
**EMT**	**BMP2**, **BMP7**, **FOXC2**, **MAP1B**, **SNAI2**, **SOX10**, **SPARC**, **SPP1**, TGFB2, **VCAN**, **ZEB1**, **WNT11**	**WNT5A**	**BMP2**, **SNAI2**, **SOX10**, **SPARC**, **VCAN**, **ZEB1**	**BMP7**, **SPP1**, **TGFB2**, **WNT5A**	**BMP2**, **FOXC2**, **MAP1B**, **SNAI2**, **SPARC**, **SPP1**, TGFB2, **ZEB1**, **WNT11**	**ITGA5**, **SOX10**, **WNT5A**	**SPARC**, **VCAN**	BMP2, **BMP7**, **ITGA5**, **SOX10**, **SPP1**, **TGFB2**, WNT11
**Stem Cell Transcriptional Factors**	**GLI2**, HOXA7, **HOXB1**, **HOXB3**, **HOXB5**, **HOXB8**, **HTR7**, **KLF4**, **NANOG**, **OLIG2**, **POU4F2**, **POU5F1**, **TBX5**	PAX6, POU4F1, **SOX2**, SOX6, ZFPM2	**EGR3**, **GLI2**, **HOXB1**, **HOXB3**, **HOXB8**, **HTR7**, **KLF4**, **LMX1B**, **NANOG**, **OLIG2**, **PAX5**, **PAX6**, **POU5F1**, **SOX6**, **TBX5**, **TDGF1**, WT1	HOXA7, **HOXB5**, **MSX2**, POU4F1, **SOX2**	**GLI2**, **HOXB3**, **HOXB5**, **HTR7**, **KLF4**, **LMX1B**, **NANOG**, **PAX5**, **POU4F2**, **POU5F1**, **SOX2**, **SOX6**, **TDGF1**, ZFPM2	**MSX2**	**EGR3**, **NANOG**, **PAX6**, POU4F1, **SOX2**	**HOXB5**, HOXB8, HTR7, TDGF1, WT1
**Tumor Metastasis**	**IGF1**, IL18, **MCAM**	—	**CXCR2**, **IGF1**, **KISS1**, **MMP10**, MMP7	IL18	**CXCR2**, **CXCR4**, **IGF1**, **MCAM**, **MMP10**, **MMP13**	—	**CXCR2**, **CXCR4**, **IGF1**, **KISS1**, KISS1R, **MMP10**, **MMP13**	—
**Angiogenesis**	**ANPEP**, **CDH5**, **CTGF**, **CXCL9**, **FGF1**, **FLT1**, **IL8**, **KDR**, **PECAM1**, **PLAU**	**TIE1**	**ANPEP**, **CTGF**, **CXCL9**, **CXCL10**, **EFNA1**, **FGF1**, **IL8**, **PLAU**, **S1PR1**, **TEK**	**ANGPT1**, **FLT1**, KDR, **PECAM1**, **TIE1**	**CDH5**, **CXCL9**, **CXCL10**, **EFNA1**, **FLT1**, **S1PR1**, **TEK**, **TIE1**	**ANGPT1**, ANPEP, **PECAM1**	**CTGF**, **CXCL10**, **EFNA1**, **IL8**, **KDR**, **PECAM1**, **S1PR1**, **TEK**, **TIE1**	**FLT1**

RT^2 Profiler PCR Arrays were analyzed. Genes that were up-regulated or down-regulated in two or more cell lines are indicated in bold letters, respectively.

Supplementary Table 2: Expression profile of miRNAs in breast cancer cell lines transfected with the PRDM14 vector or PRDM14 shRNA

Breast cancer cell lines transfected with the PRDM14 vector					
**miRNA**	**fold change**	**MCF7 - PRDM14+**	**MDA-MB-231 - PRDM14+**	**HCC1937 - PRDM14+**	**MCF 10A - PRDM14+**
**Up-regulated**	> 10.0	miR-10a, miR-155, miR-221	—	—	miR-582-3p
	> 1.5	miR-101, miR-130a, miR-135a, miR-149, miR-21, miR-222, miR-23a, miR-23b, miR-29a, miR-32, miR-502, miR-520g, miR-548d-5p, miR-582-3p, miR-98	miR-101, miR-125a-3p, miR-130a, miR-149, miR-155, miR-184, miR-21, miR-215, miR-23a, miR-23b, miR-502, miR-548d-5p, miR-582-3p, miR-98	miR-101, miR-184, miR-23b, miR-323-3p, miR-502, miR-519d, miR-519e, miR-542-5p, miR-582-3p	miR-125a-3p, miR-149, miR-184, miR-21, miR-215, miR-32, miR-34c, miR-486-3p, miR-519e, miR-548d-5p, miR-627
**Down-regulated**	< 0.5	miR-125a-3p, miR-200b, miR-323-3p, miR-485-3p, miR-486-3p, miR-542-5p, miR-627	miR-128a, miR-15a, miR-200a, miR-200b, miR-323-3p, miR-485-3p, miR-486-3p, miR-503, miR-519d, miR-519e, miR-520f, miR-520g, miR-627	miR-10a, miR-128a, miR-222, miR-29a, miR-503, miR-520f	miR-23b
	< 0.1	miR-519d	miR-34c	miR-34c, miR-627	miR-193a-3p, miR-200a, miR-502

Breast cancer cell lines transfected with PRDM14 shRNA			
**miRNA**	**fold change**	**MCF7 - PRDM14 shRNA**	**HCC1937 - PRDM14 shRNA**
**Up-regulated**	> 10.0	miR-138, miR-150, miR-193a-3p, miR-199a-3p, miR-29b, miR-331-5p, miR-502-3p	let-7c, miR-150, miR-15a, miR-296, miR-339-5p, miR-34a, miR-375, miR-411, miR-489, miR-517b
	> 1.5	miR-139-5p, miR-148b, miR-193a-5p, miR-296	miR-128a, miR-148b, miR-183, miR-148b, miR-183, miR-199a-3p, miR-203, miR-29a, miR-301, miR-302a, miR-340
**Down-regulated**	< 0.5	miR-212, miR-221, miR-25, miR-27b, miR-301, miR-483-5p, miR-485-3p, miR-501, miR-625	miR-103, miR-106a, miR-125a-3p, miR-139-5p, miR-149, miR-186, miR-193a-3p, miR-193a-5p, miR-195, miR-211, miR-212, miR-215, miR-218, miR-23a, miR-24, miR-27a, miR-27b, miR-28, miR-28-3p, miR-32, miR-422a, miR-483-5p, miR-485-3p, miR-501, miR-542-3p, miR-625
	< 0.1	miR-205, miR-23a, miR-450b-5p, miR-493, miR-500	miR-107, miR-130a, miR-130b, miR-135b, miR-138, miR-18a, miR-18b, miR-205, miR-221, miR-222, miR-224, miR-29b, miR-331-5p, miR-450b-5p, miR-500, miR-576-5p, miR-642, miR-99a

miRNAs that were up-regulated or down-regulated in two or more cell lines are indicated in red or blue, respectively.

Supplementary Table 3: Epigenome-wide DNA methylation analyses of breast cancer cells transfected with a PRDM14 expression vector or a PRDM14 shRNA compared with control vector-transfected cells

	Equally methylated regions (Log ratio > 0.6)	Differentially methylated regions (Log ratio > 0.6)		Hyper methylated/ Differentially methylated (%)	Hypo methylated/ Differentially methylated (%)	Differentially methylated/ Equally methylated (%)
		Hyper methylated	Hypo methylated			
**HCC1937 PRDM14+**	26036	39	157	19.9	80.1	0.75
**MCF7 PRDM14+**	26425	63	104	37.7	62.3	0.63
**MCF-10A PRDM14+**	26206	329	57	85.2	14.8	1.47
**MDA-MB-231 PRDM14+**	24749	1346	497	73	27	7.45
**HCC1937 shRNA PRDM14**	19788	3609	3209	52.9	47.1	34.5
**MCF7 shRNA PRDM14**	25476	596	534	52.7	47.3	4.44

A Human Methylation 450 BeadChip array with 96% of CpG islands was used for these analyses. The presented data are specific for genomic regions with "high CpGs" (Functional annotation of the mammalian genome [FANTOM 4] promoters with high CpG content).

Supplementary Table 4: Genes with bivalent chromatin occupied by PRDM14

Gene	Gene Function	Reference(s)
**Common genes in both MDA-MB-231 and HCC1937 cells**		
**HOMER2**	Anchorage-independent growth	[62]
**SH3GL3**	Invasion and metastasis of glioma and oral SCC	[63, 64]
**MDA-MB-231**		
**NOTCH2**	Related to cancer stemness	[65]
**S100A2–A6 cluster**	Promote invasion and angiogenesis	[66, 67]
**LAMC1/2**	Cell adhesion, differentiation, migration, metastasis, and angiogenesis	[68]
**SRGAP2**	Cell spreading and cell migration	[69]
**ACTN2**	Cell proliferation and metastasis	[70, 71]
**PIWIL4**	Maintenance of germline stem cells and distant cancer metastasis; highly expressed in breast cancer tissues; enhance cell migration; resistance to apoptosis	[72, 73]
**RAB3IP**	Interact with cancer-related protein SSX2	[74]
**HS6ST3**	Proliferation, differentiation, adhesion, and migration	[75]
**RAD51B**	Interact with BRCA2	[76]
**NEDD4**	Proto-oncogenic ubiquitin ligase for PTEN	[77]
**CSNK1G1**	Promote Wnt/β-catenin signaling	[78]
**NFAT5**	Enhance cell migration	[79]
**STAT3**	Cancer initiation and progression	[80]
**MAP3K3**	Resistance to apoptosis through activation of NF-κB	[81]
**MAPRE2**	Involved in perineural invasion	[82]
**HCC1937**		
**CAMK4**	Resistance of hydrogen peroxide-induced apoptosis	[83]
**CASC4**	Associated with HER-2 overexpression	[84]
**DMXL2**	Amplify Notch signaling	[85]
**PECAM1**	Tumor cell adhesion to endothelium	[86]
**DACH2**	Poor prognosis marker in ovarian cancer	[87]
**ENOX2**	Enhance cell migration and EMT; essential for growth of early embryos but absent from normal adult cells; expressed in cancer	[88, 89]

EMT, Epithelial-Mesenchymal Transition.

Supplementary Table 5: Sequences of siRNAs and shRNAs used in this study

	First base	Sequence	Region
**shRNA#1**	1087	CTACGGAGACAATTCTGTG	CDS
**shRNA#3**	1299	ATGGAGACTGCTATGAGAA	CDS
**Control siRNA**	Scrambled		–
**siRNA#2**	1082	AAGACCTACGGAGACAATTCTGT	CDS
**siRNA#3**	1504	TTCTCTCTGCAAACGATCCTTTG	CDS
**siRNA#5**	2268	GAGATTCGGATTCTCTCTATTTC	3′UTR

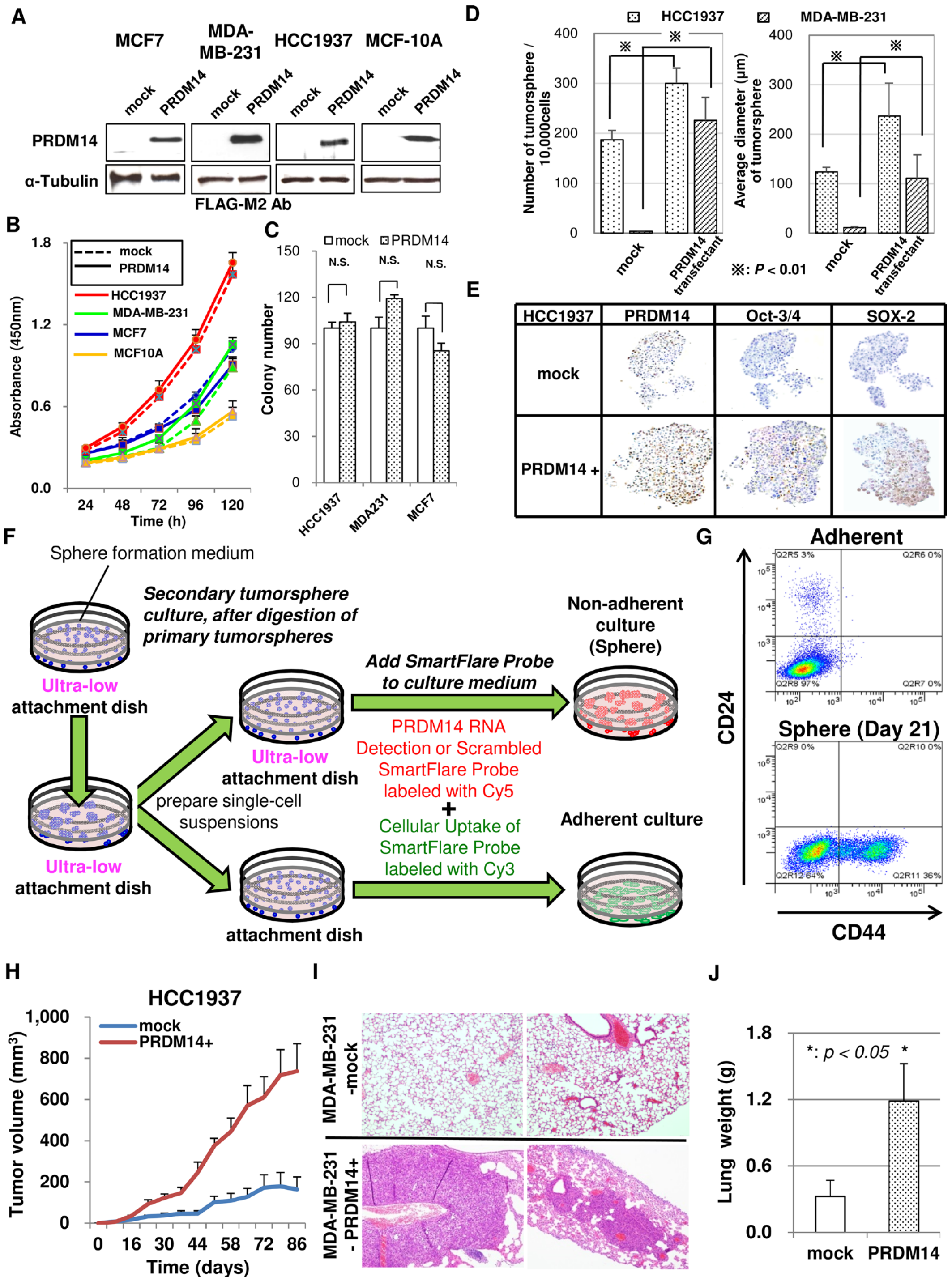

Supplementary Figure 1: PRDM14 is required for the stemness phenotype of cancer cells (related to Figure 2). (**A**) PRDM14 expression in whole-cell extracts prepared from human breast cancer cells transfected with PRDM14-FLAG. α-Tubulin served as the loading control. (**B**) Viabilities of PRDM14-transfected breast cancer and MCF-10A cells were determined using a WST-8 assay. Experiments were performed in triplicate (n = 6). (**C**) Colony formation assay of breast cancer cell lines. Data are presented as mean ± standard deviation (n = 3). (**D**) Tumorspheres formed by PRDM14-transfected HCC1937 and MDA-MB-231 cells. The number and diameter of tumorspheres formed per 10,000 cells on day 14 are shown. ※P<0.01, compared to mock-transfected controls. (**E**) Immunohistochemistry expression analysis of PRDM14, Oct-3/4, and SOX-2 in paraffin-embedded tumorspheres of PRDM14-transfected HCC1937 cells. (**F**) Flowchart for detecting *PRDM14* mRNA using SmartFlare probes in primary breast cancer cells cultured under tumorsphere (non-adherent) or adherent conditions. (**G**) Flow cytometry of primary breast tumor cells under tumorsphere (non-adherent) or adherent conditions. (**H**) PRDM14-transfected HCC1937 cells were injected into the mammary fat pads of nude mice and tumor growth was measured *in vivo*. Data are expressed as mean ± standard deviation (n = 10). (**I**, **J**) Lung metastases formed by engrafted MDA-MB-231 cells transfected with a PRDM14 expression vector. (I) Sections of lung tissues stained with hematoxylin and eosin. 10× magnification. (J) Lung weight. Data are expressed as mean ± standard deviation (n = 8).

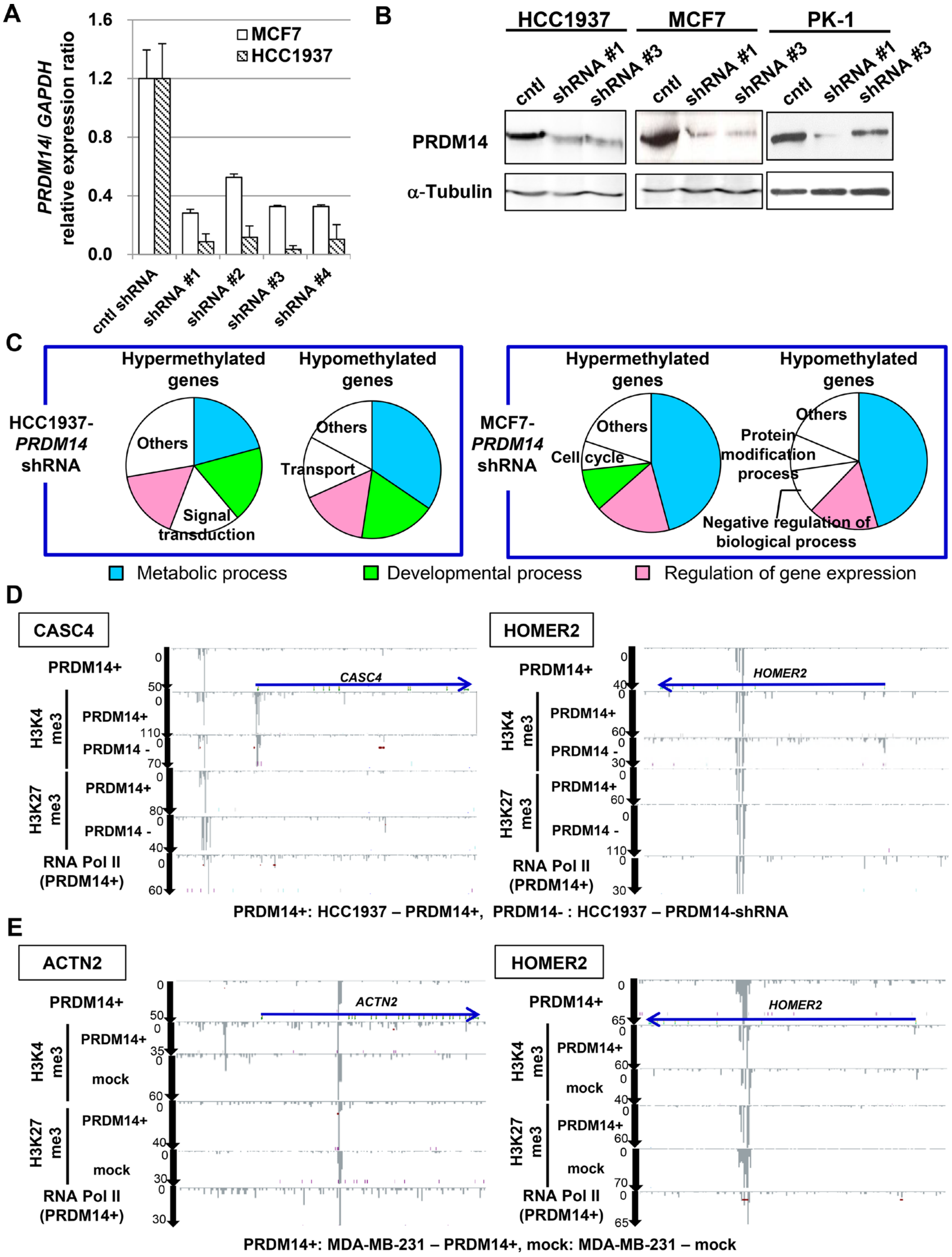

Supplementary Figure 2: PRDM14 expression levels of cells transfected with a PRDM14-shRNA and PRDM14-induced epigenetic changes in the genome (related to Figures 3 and 4). (**A**) qRT-PCR analysis for *PRDM14* expression levels in cells transfected with a *PRDM14* shRNA. PRDM14 expression levels of the HCC1937 and MCF7 cell line negative controls were adjusted to the value of the MCF7 control. Data are expressed as mean ± standard deviation (*n* = 3). (**B**) Immunoblot analyses of PRDM14 and α-tubulin in cells transfected with a *PRDM14* shRNA. The pancreatic cancer cell line PK-1 was included for comparative purposes. (**C**) Gene ontology analyses of genes with hypermethylated or hypomethylated regions using HCC1937 and MCF7 cells transfected with the PRDM14 shRNA. (D, E) Effect of PRDM14 expression on PRDM14-occupied genes and histone modifications. The x- and y-axes correspond, respectively, to genomic location and depth of coverage based on strand-specific sequence read numbers obtained after subtracting the background. Profiles of (**D**) PRDM14-transfected (PRDM14+) or shRNA-transfected (PRDM14–) HCC1937 cells and (**E**) PRDM14-transfected (PRDM14+) or control vector-transfected (mock) MDA-MB-231 cells.

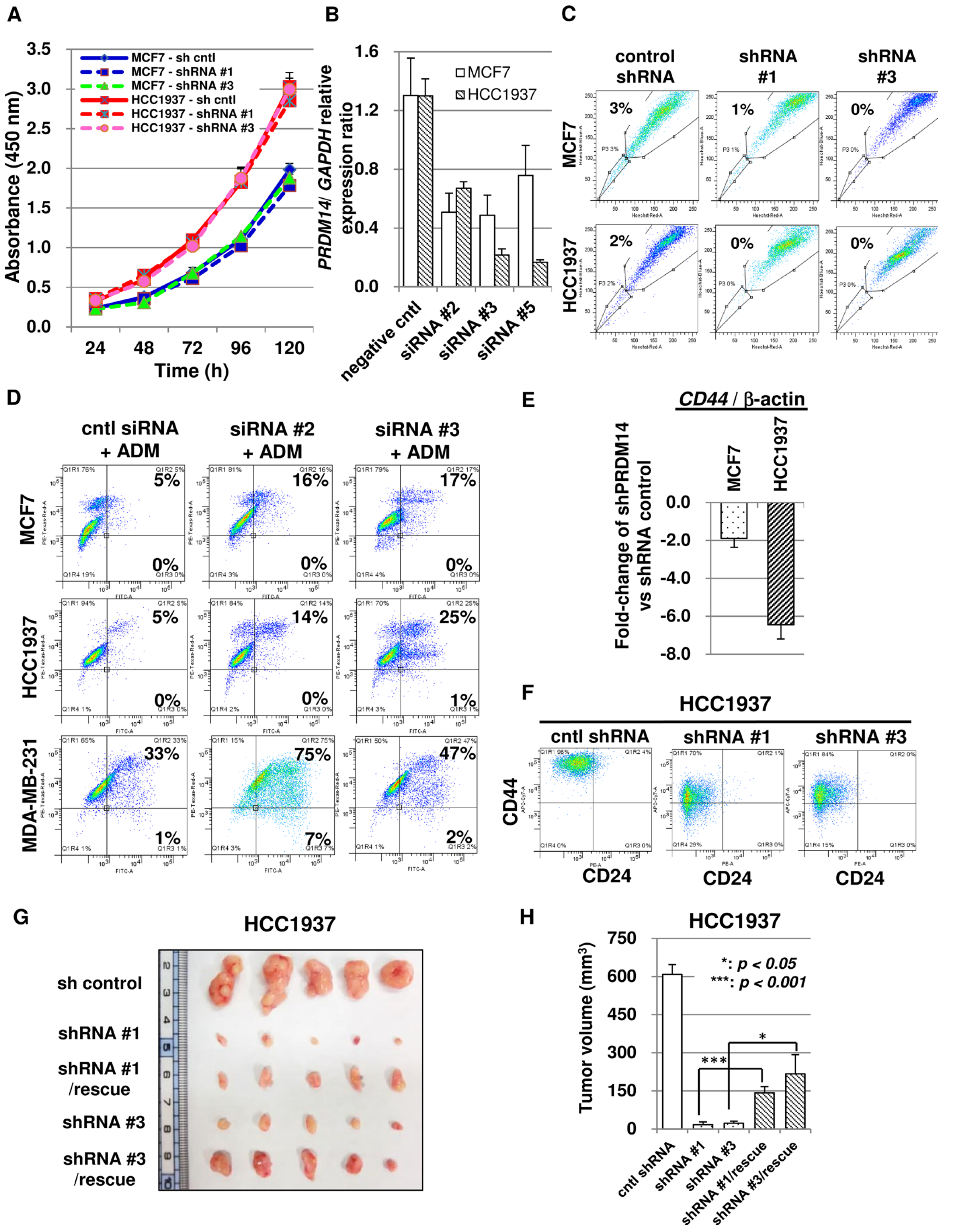

Supplementary Figure 3: Decreasing PRDM14 expression eliminates the CSC population of human cancer cell lines (related to Figure 4). (**A**) Effect of decreasing the expression of PRDM14 with shRNA on the viability of breast cancer cells. WST-8 assays were performed in triplicate. Data are expressed as mean ± standard deviation (n = 6). (**B**) *PRDM14* expression levels of siRNA transfectants. PRDM14 expression levels of the HCC1937 and MCF7 cell line negative controls were adjusted to the value of the MCF7 control. Data are expressed as mean ± standard deviation (n = 3). (**C**) Side-population fraction of MCF7 and HCC1937 cells stably transfected with a PRDM14-shRNA expression vector. (**D**) Effects of PRDM14 on apoptosis of breast cancer cell lines transfected with a PRDM14-siRNA and exposed to anticancer drugs. The cells were treated with annexin V and propidium iodide. The lower and upper right quadrants indicate annexin-positive early apoptotic cells and annexin/propidium iodide-positive late apoptotic cells, respectively. (E–F) Effect of PRDM14 on the expression of CD44 by human breast cancer cells. (**E**) qRT-PCR analysis of *CD44* expression by PRDM14 knockdown in HCC1937 cells. Data are expressed as mean ± standard deviation (n = 3). (**F**) FACS analyses of CD24 and CD44 expression by PRDM14 knockdown in HCC1937 cells (G–H). Effect of PRDM14 re-expression on tumor morphologies (**G**) and volumes (**H**) at the end point (day 42). Data are expressed as mean ± standard deviation (n = 8).

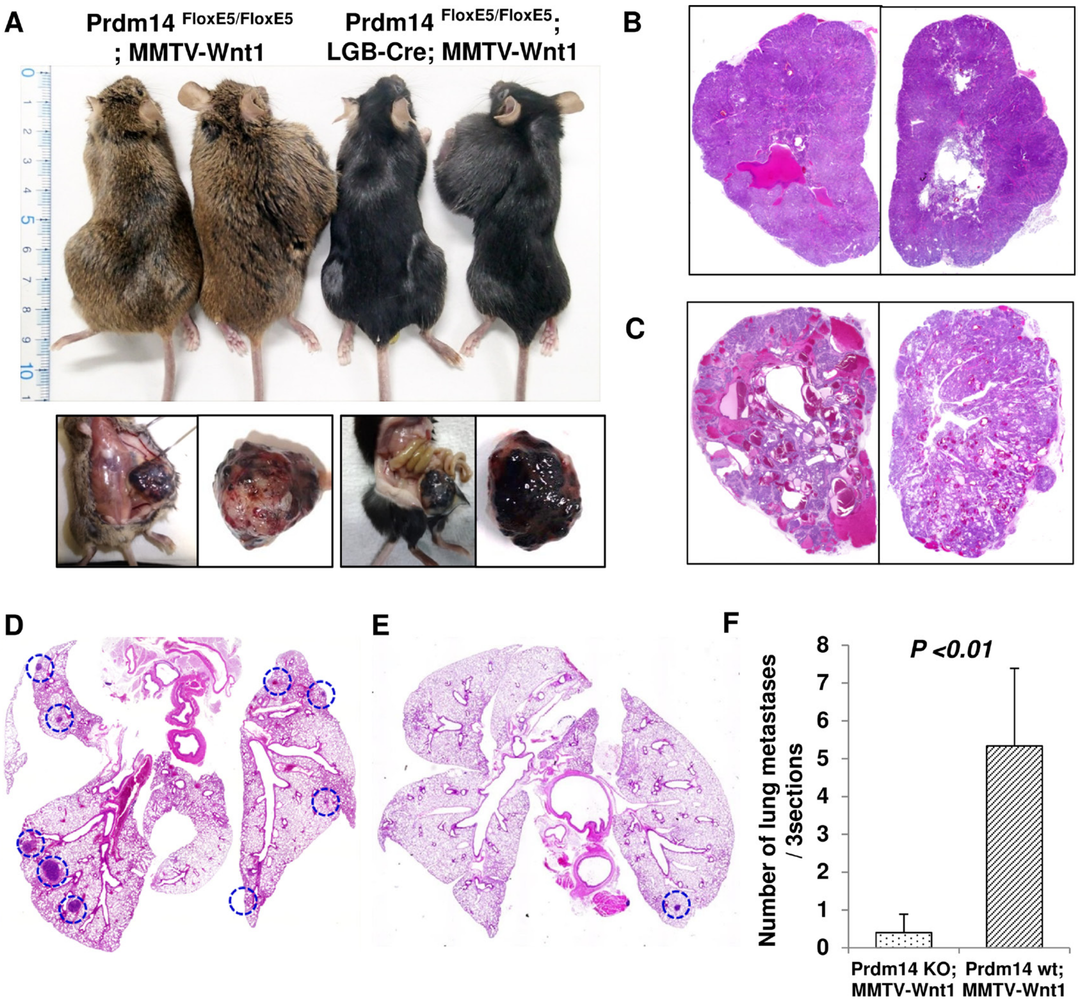

Supplementary Figure 4: Prdm14 knock out improved disease-free survival in MMTV-Wnt1 transgenic mice and reduced spontaneous lung metastasis (related to Figure 5). (**A**) Representative image of Prdm14$^{flox/flox}$; Wnt-1 mice, and Prdm14$^{flox/flox}$; LGB-Cre; Wnt-1 transgenic (Prdm14 KO; Wnt-1 transgenic) mice. (**B**, **C**) Representative image of spontaneous breast tumor in (B) Prdm14$^{flox/flox}$; Wnt-1 mice, and (C) Prdm14 KO; Wnt-1 transgenic mice (**D**–**F**). Suppression of spontaneous lung metastases in Prdm14 KO; Wnt-1 transgenic mice compared with Prdm14$^{flox/flox}$; Wnt-1 mice. Representative images of histological lung sections from (D) Prdm14$^{flox/flox}$; Wnt-1 mice or (E) Prdm14 KO; Wnt-1 transgenic mice. Spontaneous lung metastatic tumors are circled with dotted blue lines. H&E staining; ×40 magnification. (F) Mean number of spontaneous lung metastases in Prdm14 KO; Wnt-1 transgenic mice and Prdm14$^{flox/flox}$; Wnt-1 mice (error bars indicate SD).
